# Ketamine Prevents Inflammation-Induced Reduction of Human Hippocampal Neurogenesis via Inhibiting the Production of Neurotoxic Metabolites of the Kynurenine Pathway

**DOI:** 10.1093/ijnp/pyae041

**Published:** 2024-09-19

**Authors:** Gargi Mandal, Madeline Kirkpatrick, Silvia Alboni, Nicole Mariani, Carmine M Pariante, Alessandra Borsini

**Affiliations:** Stress, Psychiatry and Immunology Laboratory, Institute of Psychiatry, Psychology and Neuroscience, Department of Psychological Medicine, King’s College London, UK; Stress, Psychiatry and Immunology Laboratory, Institute of Psychiatry, Psychology and Neuroscience, Department of Psychological Medicine, King’s College London, UK; Department of Life Sciences, University of Modena and Reggio Emilia, Modena, Italy; Stress, Psychiatry and Immunology Laboratory, Institute of Psychiatry, Psychology and Neuroscience, Department of Psychological Medicine, King’s College London, UK; Stress, Psychiatry and Immunology Laboratory, Institute of Psychiatry, Psychology and Neuroscience, Department of Psychological Medicine, King’s College London, UK; Stress, Psychiatry and Immunology Laboratory, Institute of Psychiatry, Psychology and Neuroscience, Department of Psychological Medicine, King’s College London, UK

**Keywords:** Cytokines, kynurenine pathway, hippocampal neurogenesis, apoptosis

## Abstract

**Background:**

Understanding the precise mechanisms of ketamine is crucial for replicating its rapid antidepressant effects without inducing psychomimetic changes. Here, we explore whether the antidepressant-like effects of ketamine enantiomers are underscored by protection against cytokine-induced reductions in hippocampal neurogenesis and activation of the neurotoxic kynurenine pathway in our well-established in vitro model of depression in a dish.

**Methods:**

We used the fetal hippocampal progenitor cell line (HPC0A07/03C) to investigate ketamine’s impact on cytokine-induced reductions in neurogenesis in vitro. Cells were treated with interleukin- 1beta (IL-1b) (10 ng/mL) or IL-6 (50 pg/mL), alone or in combination with ketamine enantiomers arketamine (R-ketamine, 400 nM) or esketamine (S-ketamine, 400 nM) or antidepressants sertraline (1 mM) or venlafaxine (1 mM).

**Results:**

Resembling the effect of antidepressants, both ketamine enantiomers prevented IL-1b– and IL-6–induced reduction in neurogenesis and increase in apoptosis. This was mediated by inhibition of IL-1b–induced production of IL-2 and IL-13 by R-ketamine and of IL-1b–induced tumor necrosis factor-alpha by S-ketamine. Likewise, R-ketamine inhibited IL-6–induced production of IL-13, whereas S-ketamine inhibited IL-6–induced IL-1b and IL-8. Moreover, both R- and S-ketamine prevented IL-1b–induced increases in indoleamine 2,3-dioxygenase expression as well as kynurenine production, which in turn was shown to mediate the detrimental effects of IL-1b on neurogenesis and apoptosis. In contrast, neither R- nor S-ketamine prevented IL-6–induced kynurenine pathway activation.

**Conclusions:**

Results suggest that R- and S-ketamine have pro-neurogenic and anti-inflammatory properties; however, this is mediated by inhibition of the kynurenine pathway only in the context of IL-1b. Overall, this study enhances our understanding of the mechanisms underlying ketamine’s antidepressant effects in the context of different inflammatory phenotypes, ultimately leading to the development of more effective, personalized therapeutic approaches for patients suffering from depression.

**Significance Statement**

Several studies have shown that ketamine is a fast-acting, efficient antidepressant that alleviates symptoms even in those suffering with treatment-resistant depression. However, its mechanisms of action are unclear. In this study we demonstrate, for the first time (to our knowledge), that treatment in vitro of human hippocampal progenitor cells with R-ketamine or S-ketamine prevents the reduction in neurogenesis caused by IL-6 and IL-1b. Additionally, our results suggest that this is achieved via the ability of the ketamine enantiomers to counteract production of specific pro-inflammatory molecules, although with some enantiomer-specific effects. However, we observe that the neuroprotective effect of ketamine enantiomers only involves inhibition of the kynurenine pathway in the context of IL-1b, highlighting the importance of stratifying patients according to their unique inflammatory profile. Overall, our findings have important implications toward the search for new and more personalized antidepressant treatment, with the aim of developing new therapeutic approaches that target the same anti-inflammatory pathways as ketamine but avoid the psychomimetic and addictive side effects.

## INTRODUCTION

In recent years, seminal research establishing the potent antidepressant properties of ketamine has led to important advancements in the search for new depression therapeutics (Berman et al., 2000). Ketamine is a noncompetitive antagonist to the N-methyl-D-aspartate receptor (NMDAR) shown in numerous studies to alleviate depressive symptoms, even in those with treatment-resistant depression (Diazgranados et al., 2010; Murrough et al., 2013; Bobo et al., 2016). Additionally, unlike classic antidepressants, ketamine’s effects are rapid, producing benefits as early as several hours after administration (Berman et al., 2000; Matveychuk et al., 2020). However, the use of ketamine could have adverse outcomes due to its addictive properties and psychotomimetic effects (Strong and Kabbaj, 2018). Therefore, uncovering the molecular mechanisms through which ketamine induces antidepressant effects is vital to developing targeted therapeutics with the same efficacy but without the pertinent limitations.

In clinical trials, ketamine is used in 1 of 2 formulations: racemic ketamine, which is a mixture of the 2 enantiomers esketamine (S-ketamine) and arketamine (R-ketamine) given intravenously; and a nasal spray containing only S-ketamine (Jelen et al., 2021). A recent meta-analysis of both ketamine formulations revealed that racemic ketamine is a more effective antidepressant than S-ketamine (Nikolin et al., 2023), indicating that R-ketamine also has antidepressant effects.

While ketamine’s action as an NMDAR antagonist is believed to be partially responsible for its antidepressant properties (Autry et al., 2011; Jelen et al., 2021), not all NMDAR antagonists possess antidepressant capabilities (Newport et al., 2015), highlighting that ketamine’s mechanism of action is likely far more complex. Additionally, preclinical evidence suggests that R-ketamine produces longer-lasting antidepressant-like effects than S-ketamine, despite it being a less potent NMDAR antagonist (Yang et al., 2015a; Fukumoto et al., 2017; Chang et al., 2019; Jelen et al., 2021). Several preclinical and clinical studies show that ketamine can decrease levels of proinflammatory cytokines, highlighting an anti-inflammatory mechanism (Beilin et al., 2007; Loix et al., 2011; Nikkheslat, 2021). This is particularly interesting given the established association between inflammation and depressive symptoms (Beurel et al., 2020; Pitharouli et al., 2021). Indeed, the decrease in serum levels of cytokines following ketamine treatment is correlated with an alleviation of depressive symptoms (Chen et al., 2018; Kopra et al., 2021).

Both animal models and clinical studies demonstrate an increased inflammatory milieu associated with depression, including increased levels of interleukin-1beta (IL-1b), IL-6, tumor necrosis factor-alpha (TNFa), and interferon-gamma (IFN-g) (Su et al., 2019; Osimo et al., 2020; Das et al., 2021). Notably, high levels of inflammatory markers are prominent in treatment-resistant depression, and the levels of cytokines can predict nonresponse to conventional antidepressants (Cattaneo et al., 2013; Kiraly et al., 2017; Yang et al., 2019). Among other mechanisms, cytokines may influence depression pathogenesis via inhibition of hippocampal neurogenesis (Wu and Zhang, 2023). Hippocampal neurogenesis, which is defined as the generation of new neurons within the subgranular zone of the dentate gyrus, has been suggested in rodent studies to be required for antidepressant efficacy (Santarelli et al., 2003). To measure hippocampal neurogenesis in vitro, we use markers to determine differentiation into immature (doublecortin [DCX]) (Gómez-Climent et al., 2008) and mature (microtubule-associated protein 2 [MAP2]) neurons (DeGiosio et al., 2022). Indeed, studies from our group have demonstrated that inflammatory cytokines decrease hippocampal progenitor cell (HPC) differentiation in vitro in our well-established cellular model of depression in a dish (Zunszain et al., 2012; Borsini et al., 2017, 2018, 2019, 2020a, 2020b, 2021, 2022). In particular, inflammation is thought to reduce neurogenesis and promote depressive symptoms via activation of the kynurenine pathway, increasing production of neurotoxic kynurenine pathway metabolites (Zunszain et al., 2012; Borsini et al., 2017; Savitz, 2020).

**Figure 1.**
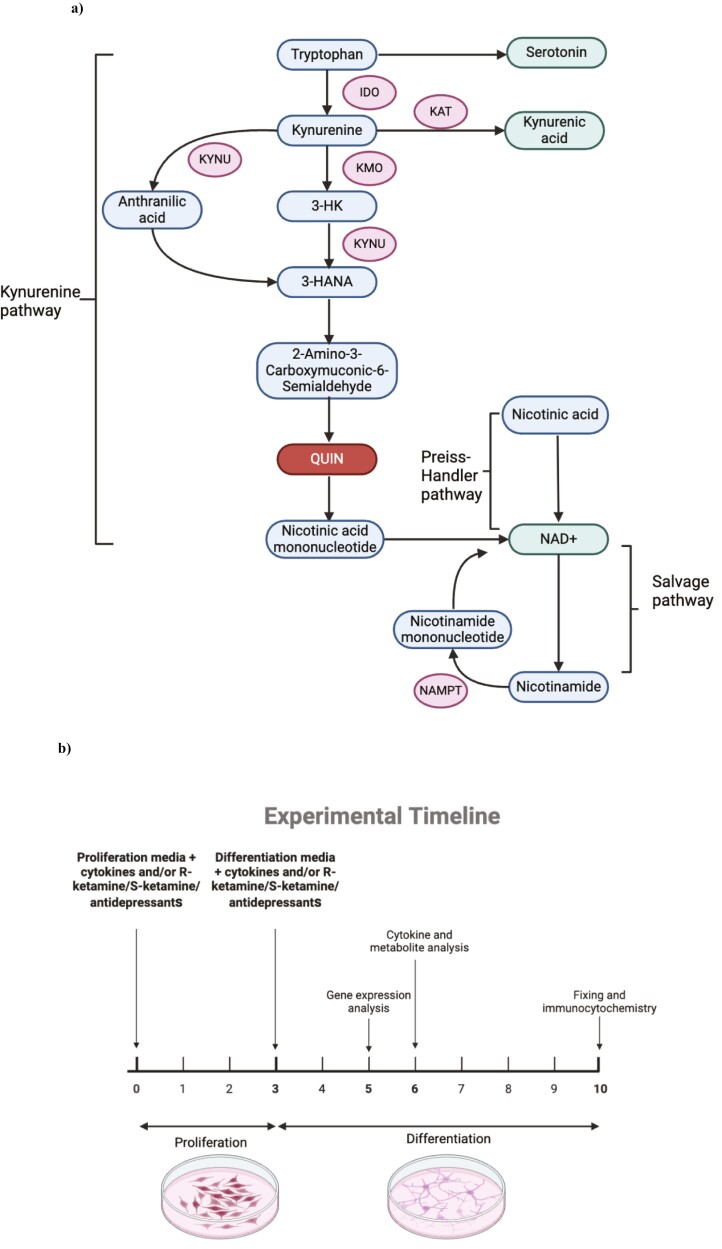
The kynurenine pathway of tryptophan metabolism (A) and timeline of cellular experiments (B). Simplified kynurenine pathway of tryptophan metabolism leading to production of either neurotoxic or neuroprotective metabolites (A); the experimental timeline used in this study: cells were first treated for 3 days with reduced modified media with growth factors (proliferation media) with or without IL-1β or IL-6 and/or R-ketamine, S-ketamine, sertraline or venlafaxine. After 3 days, media was removed, and cells were given media without growth factors (differentiation media) and treatment with all compounds continued. For analysis of gene expression, RNA was isolated after 2 days in differentiation. For analysis of cytokines and kynurenine pathway metabolites, supernatant was collected after 3 days in differentiation. For analysis of differentiation and apoptosis, cells were fixed after 7 days of differentiation (10 days total treatment) and immunocytochemistry was performed (B). Abbreviations: 3-HANA, 3-hydroxyanthranilic acid; 3-HK, 3-Hydorxykynurenine; IDO, indolamine-2,3,dioxygenase; KAT, kynurenine aminotransferase; KMO, kynurenine-3-monooxygenase; KYNU, kynureninase; NAD+, nicotinamide adenine dinucleotide; NAMPT, nicotinamide phosphoribosyl transferase; QUIN, quinolinic acid.

The kynurenine pathway begins when tryptophan, a serotonin precursor, is degraded into kynurenine under regulation of indoleamine 2,3-dioxygenase (IDO) (Figure 1). Inflammatory cytokines, such as IL-1b, IL-6, TNF-a, and IFN-g, can induce IDO and subsequently promote degradation of tryptophan down the kynurenine pathway (Kwidzinski and Bechmann, 2007; O’Connor et al., 2009; Zunszain et al., 2012; Fischer et al., 2015; Borsini et al., 2017). Additionally, IL-1b activates the enzymes kynurenine-3-monooxygenase (KMO) and kynureninase (KYNU), which are involved in the production of further kynurenine pathway metabolites, such as anthranilic acid, 3-hydroxykynrenine acid (3-HK), and eventually the neurotoxic quinolinic acid (QUIN) (Figure 1) (Zunszain et al., 2012). Under physiological conditions, production of QUIN along the kynurenine pathway is used to produce de novo NAD+ from tryptophan. Indeed, QUIN is the endogenous source of nicotinamide (NIC) and NAD+ (Savitz, 2020). In addition to de novo synthesis, NAD+ can be produced from nicotinic acid (NICA) via the Preiss-Handler pathway or from NIC via the salvage pathway (Fricker et al., 2018; Castro-Portuguez and Sutphin, 2020).

In addition to its role in NAD+ production, QUIN also behaves as an NMDA receptor agonist, inducing neurotoxicity via glutamate excitotoxicity and ultimately resulting in reduced hippocampal neurogenesis (Lugo-Huitrón et al., 2013). Alternatively, kynurenine can be metabolized via kynurenine aminotransferase into kynurenic acid (KYNA), which has neuroprotective properties. Interestingly, a review of clinical studies (Kopra et al., 2021) has revealed that treatment of depressed patients with ketamine can modulate levels of kynurenine pathway metabolites, with many commonly establishing that ketamine treatment decreases kynurenine levels (Zhou et al., 2018; Kopra et al., 2021). This highlights that ketamine’s anti-inflammatory properties may allow it to inhibit kynurenine pathway activation and thus provide neuroprotection.

Previous studies from our group have established that antidepressant compounds can prevent cytokine-induced reductions in HPC neurogenesis via decreased production of neurotoxic kynurenine pathway components (Borsini et al., 2017). However, it is unknown whether ketamine can modulate the effect of cytokines on HPCs in a similar manner. Therefore, in this study we aimed to determine if treatment of HPCs in vitro with R-ketamine or S-ketamine can prevent reductions in neurogenesis caused by cytokines and whether this involves modulation of the kynurenine pathway. Due to evidence highlighting that the different ketamine enantiomers have different antidepressant efficacies; we will treat cells with R-ketamine and S-ketamine separately. Additionally, it has been demonstrated that R-ketamine induced fewer dissociative and psychotomimetic effects compared with S-ketamine (Passie et al., 2021). To the best of our knowledge, this is the first study to directly compare the effects of the 2 ketamine enantiomers in an in vitro model of depression using human hippocampal neuronal cells.

## MATERIALS AND METHODS

### Cell Culture

For all experiments, the multipotent human HPC line HPC0A07/03C (provided by ReNeuron, Surrey, UK) was used. Cells were allowed to proliferate in reduced modified media (for details on media reagents, see Zunszain et al., 2012; Borsini et al., 2017) supplemented with the growth factors epidermal growth factor, basic fibroblast growth factor, and 4-hydroxytamoxifen (4-OHT). Cells were grown in 25-cm^2^ filtered cap culture flasks (Nunclon, Roskilde, Denmark) at 37°C in 5% CO_2_ and regulatory passaged at 80% confluence before being transferred to plates. To initiate differentiation, growth factors and 4-OHT were removed. Detailed information on this cell line can be found in our previous publications (Anacker et al., 2013a; Borsini et al., 2017, 2018, 2019, 2020a, 2020b, 2021, 2022).

### Assays With Antidepressants and Ketamine Enantiomers

For the experiments described below, treatment conditions and doses of cytokines and antidepressants were chosen as described in our previous work (Zunszain et al., 2012; Borsini et al., 2017, 2020a, 2020b). Ketamine enantiomers were selected to fall within the range of concentrations observed in the plasma of depressed patients after ketamine infusion therapy (Zarate et al., 2012; Zhao et al., 2012). Cells were plated in 96-well plates (Nunclon) at a density of 15 000 cells per well and allowed to adhere for 24 hours. Cells were then treated, for 3 days of proliferation, with either IL-1b (10 ng/mL) or IL-6 (50 pg/mL) alone or in combination with R-ketamine (400 nM), S-ketamine (400 nM), sertraline (1 mM), or venlafaxine (1 mM). After 3 days of proliferation, cell media were removed, and treatment with the cytokines and each compound was repeated in media without growth factors and 4-OHT for an additional 7 days to allow the cells to differentiate. Subsequently, cells were fixed with 4% paraformaldehyde (PFA) and immunocytochemistry was performed. RNA was extracted after 2 days of differentiation for the measurement of expression of candidate kynurenine pathway genes, whereas supernatant was collected after 3 days of differentiation for analysis of cytokine production and kynurenine pathway metabolites. Finally, to determine the specific, direct effect of each kynurenine metabolite on neurogenesis, cells were treated from day 3 until day 7 of differentiation with the same metabolites previously found to be elevated in the supernatant. See Figure 1B for an experimental timeline.

### Immunocytochemistry and Quantification of Immunofluorescence

Immunocytochemistry was performed on day 7 of differentiation to investigate changes in neurogenesis and apoptosis, DCX, MAP2, and caspase-3 (CC3). Briefly, fixed cells were incubated with blocking solution (5% normal donkey serum, Scientific Laboratory Supplies Ltd, Nottingham, UK) for 2 hours at room temperature before incubation with primary antibodies (rabbit anti-DCX, 1:500; mouse anti-MAP2 [HM], 1:500; rabbit anti-CC3 1:500) at 4°C overnight. Cells were incubated sequentially in blocking solution for 30 minutes, secondary antibodies (Alexa 488 donkey anti-rabbit; 1:1000; Alexa donkey 555 anti-mouse; 1:1000, Invitrogen, Carslbad, CA, USA) for 2 hours, and 4’,6-diamidino-2-phenylindole dye for 5 minutes. Detailed information on the immunocytochemistry procedure can be found in our previous publication (Borsini et al., 2017). The percentage of DCX-, MAP2- and CC3-positive cells over total 4’,6-diamidino-2-phenylindole–positive cells was counted using an automated approach using CellInsight NXT High content screening platform (ThermoScientific,Waltham, MA, USA) (supplementary Fig. 1A–C for representative images).

### RNA Isolation and cDNA Synthesis

RNA was isolated from cells in 6-well plates on day 2 of differentiation using the RNeasy Plus Micro Kit (Qiagen, Crawley, UK) following the manufacturer’s instructions. Samples were stored at −80°C before further use. RNA quality and quantity were assessed by evaluation of the A260/280 and A260/230 ratios using a Nanodrop spectrometer (Nanodrop Technologies, Wilmington, DE, USA). For cDNA synthesis, 1 mg of RNA was reverse transcribed using Superscript III enzyme (Invitrogen, Carslbad, CA, USA), as previously described (Borsini et al., 2018).

### Quantitative Real-Time PCR (qPCR) Analyses

qPCR was performed using Predesigned TaqMan Gene Expression Assay probes (Life Technologies, Carslbad, CA, USA) with TaqMan Universal PCR Master Mix with UNG (Life Technologies, Carslbad, CA, USA), using Chromo 4 DNA instrument from BioRad. The expression of target genes *IDO*, *KMO*, and *KYNU* was normalized to the expression levels of beta actin and glyceraldehyde-3-phosphate dehydrogenase as references. The relative expression levels of target genes detected were calculated using the Pfaffl method (Pfaffl, 2001), with data normalized to the geometric mean of the housekeeping genes and expressed as fold change compared with the control sample.

### Multiplex Cytokine Assay

The concentration of cytokines in the supernatant was measured at day 3 of differentiation. Supernatants were analyzed using the Human Proinflammatory V-Plex Panel 1 Kit from Meso Scale delivery (Gaithersburg, MD, USA) according to the manufacturer’s instructions. In brief, 50 mL of each diluted sample was added in duplicate to the Meso Scale delivery plate before shaking at 700 rpm for 2 hours at room temperature. Subsequently, the plate was washed 3 times before addition of 25 mL detection antibody to each well and shaking for another 2 hours at 700 rpm. Finally, the plate was washed 3 times, and 150 mL of read buffer was added to each well. The plate was analyzed using the SECTOR Imager machine to measure a panel of 10 cytokines: IL-1b, IL-2, IL-4, IL-6, IL-8, IL-10, IL-12, IL-13, TNF-a, and IFN-g.

### Liquid Chromatography

The concentration of kynurenine pathway metabolites was measured on day 3 of differentiation. Fifty µL of supernatants was added with an equal volume of ice-cold 1 M perchloric acid (HClO_4_) fortified with a mix of the following stable isotope-labeled internal standard (final concentration 1 µM): L-kynurenine-d4, kunurenic acid-d5 (Buchem BV), and L-Tryptophan-d5 (Sigma-Aldrich, Burlington, MA, USA). Samples were centrifuged (15 000 × g, 15 minutes), and the supernatants were collected and directly injected into liquid chromatography with tandem mass spectrometry (LC-MS/MS). The analyses of kynurenine, tryptophan (TRP), anthranilic acid (ANA), KYNA, 3-HK, 3-hydroxyanthranilic acid (3-HANA), QUIN, NICA, and NIC in the supernatant were performed using an Agilent HP 1200 liquid chromatograph (Agilent, Milan, Italy) consisting of a binary pump, an autosampler, and a thermostated column compartment. Chromatographic separations were carried out using a Discovery HS-F5 column (3-µm particle size, 150 × 2.1 mm, Supelco, Milan, Italy) using 0.1% formic acid in water andacetonitrile (ACN) as mobile phase. The high performance liquid chromatography (HPLC) analyses were carried out using a linear elution profile of 15 minutes from 5% to 90% of ACN. The column was washed with 90% ACN for 3.5 minutes then equilibrated for 5 minutes with 5% ACN. The flow rate was 0.5 mL/min. The injection volume was 20 µL. An Agilent 6410 triple quadrupole-mass spectrometer with an electrospray ion source operating in positive mode was used for detection. The SRM pairs were 205–>188, 209–>192, 138–>120, 190–>144, 123–>80and 124–>80 for TRP, KYN, ANA, KYNA, NIC, and NICA, respectively. The calibration curves were constructed using calibration standards and were linear over the concentration range of 0.0064–5.000 µM for ANA, KYNA, PIC, and NICA; 0.0128–10.00 µM for KYN, and NIC; and 0.1280–100 µM for TRP, with a correlation coefficient (r^2^) included between the values 0.9979 and 0.9991.

### Statistical Analysis

Data are presented as mean ± SEM. All statistical analyses were performed with GraphPad Prism 7 on a minimum of 6 biological replicates. Two-way ANOVA with Bonferroni post hoc test was used for multiple comparisons. *P* <.05 was considered significant.

## RESULTS

### Ketamine Enantiomers and Antidepressants Equally Prevent the IL-1b– and IL-6–Induced Reduction of Neurogenesis

**Figure 2.**
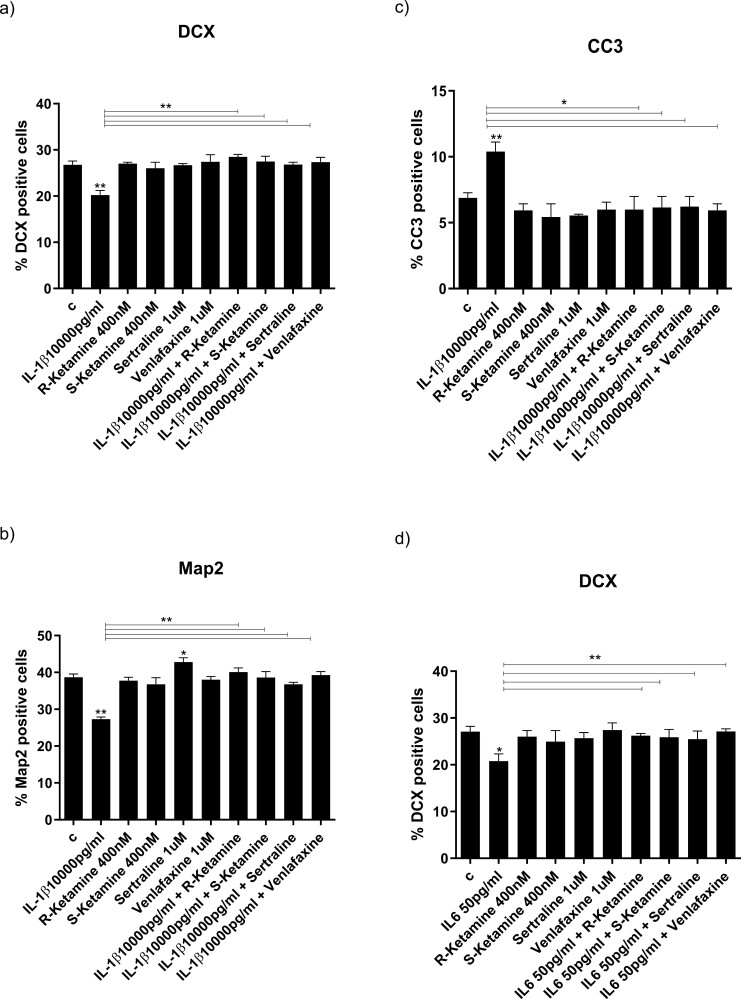

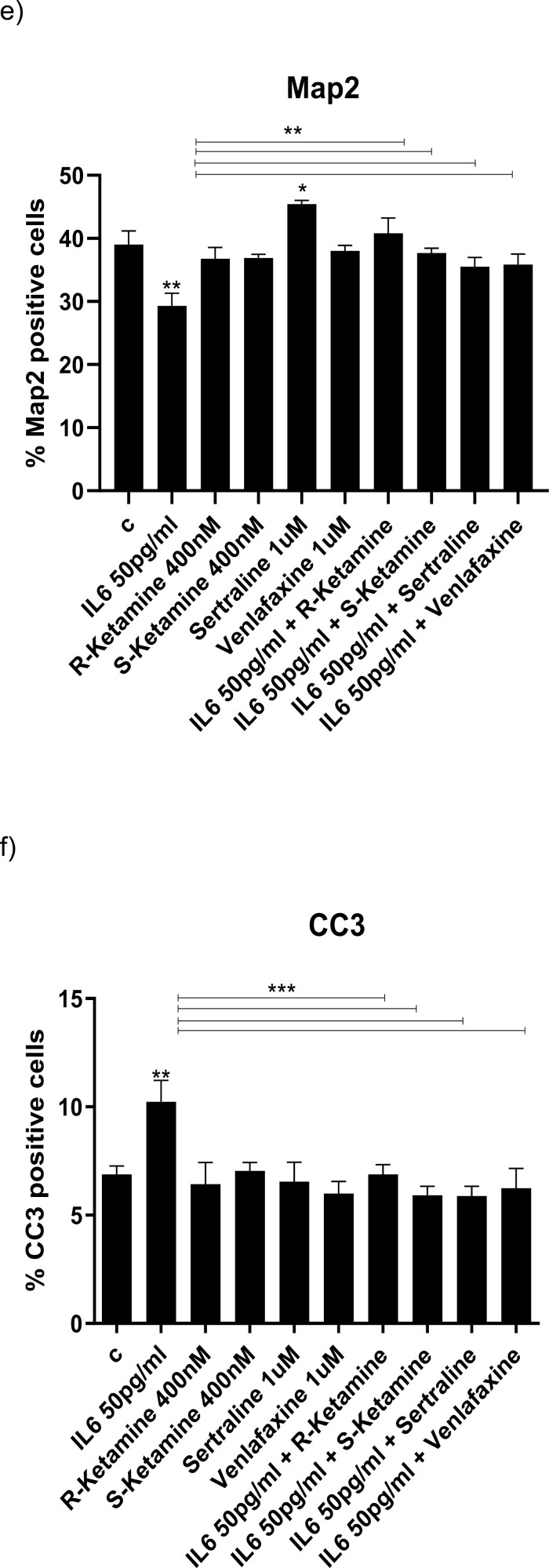
Ketamine and antidepressants reverse the IL-1b- and IL-6–induced reductions in neurogenesis and increases in apoptosis in human hippocampal progenitor cells. Treatment with IL-1b (10 000 pg/mL) decreases the number of DCX-positive (A) and MAP2-positive (B) neurons and increases the number of CC3 positive neurons (C). Treatment with R-ketamine, S-ketamine, sertraline, or venlafaxine reverse the IL-1b-induced decrease in DXC-positive cells (A) and MAP2 positive cells (B), and the increase in CC3 positive cells (C). Treatment with IL-6 (50 pg/mL) decreases the number of DCX-positive (D) and MAP2-positive (E) neurons and increases the number of CC3 positive neurons (F). Treatment with R-ketamine, S-ketamine, sertraline, or venlafaxine reverse the IL-6–induced decrease in DXC-positive cells (D) and MAP2 positive cells (E), and the increase in CC3 positive cells (F). Two-way ANOVA was performed. Data are shown as mean; *P < .05, **P < .01, ***P < .001 comparisons as indicated.

As we had previously shown (Zunszain et al., 2012), treatment of cells with IL-1b (10 ng/mL) after 7 days of differentiation resulted in decreased differentiation, demonstrated through a decrease in both early markers (numbers of DCX-positive, −6%, *P *< .01, vs vehicle; Figure 2A) and mature markers (MAP2-positive cells,−12%, *P < *.01, vs vehicle, Figure 2B), as well as increased apoptosis (increased numbers of CC3-positive cells, +4%, *P < *.01, vs vehicle; Figure 2C). As in our previous studies (Borsini et al., 2022), similar effects were observed for IL-6 (50 pg/mL) (DCX: −9%, *P < *.05, vs vehicle; Figure 2D; MAP2: −10%, *P < *.01, vs vehicle; Figure 2E; CC3: +4%, *P < *.01, vs vehicle; Figure 2F). We now add to these findings by showing that co-incubation of cells with either R-ketamine (400 nM), S-ketamine (400 nM), sertraline (1 mM), or venlafaxine (1 mM) fully prevented IL-1b–induced decrease of DCX-positive and MAP2-positive cells (Figure 2A–B) and the increase of CC3-positive cells (Figure 2C). Again, similar effects were observed for IL-6 (Figure 2D–F). Treatment with either ketamine enantiomer or venlafaxine alone did not affect the number of DCX-, MAP2-, or CC3-positive cells (Figure 2A–F), whereas, as in our previous finding (Borsini et al., 2017), sertraline alone increased the number of MAP2-positive cells (+3%, *P < *.05, vs vehicle; Figure 2B). We additionally treated the cells with IL-1β and IL-6, and R- and S-ketamine, in co-treatment (supplementary Figure 2). Results showed that there was no additive detrimental or protective effect of co-treating cells with both cytokines and ketamine enantiomers, respectively. Based on these results, and the fact that the main objective of this study was to differentiate different underlying mechanisms, we focused on the effects of treating with each cytokine or ketamine enantiomer alone. Overall, these findings are consistent with the notion that, similarly to antidepressants, R-ketamine and S-ketamine can positively affect neurogenesis when in the presence of an immune challenge, like cytokines, thereby preventing their detrimental effects of inflammation on neuronal cells.

### Ketamine Enantiomers and Antidepressants Prevent IL-1b– and IL-6–Induced Increased Production of Specific Cytokines

**Figure 3.**
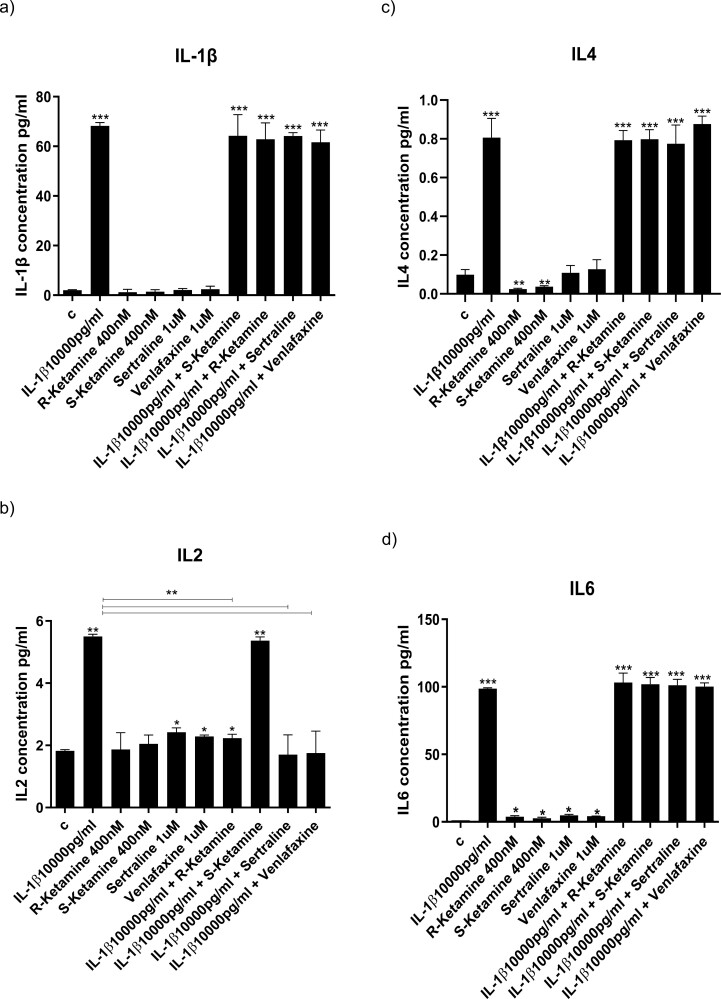

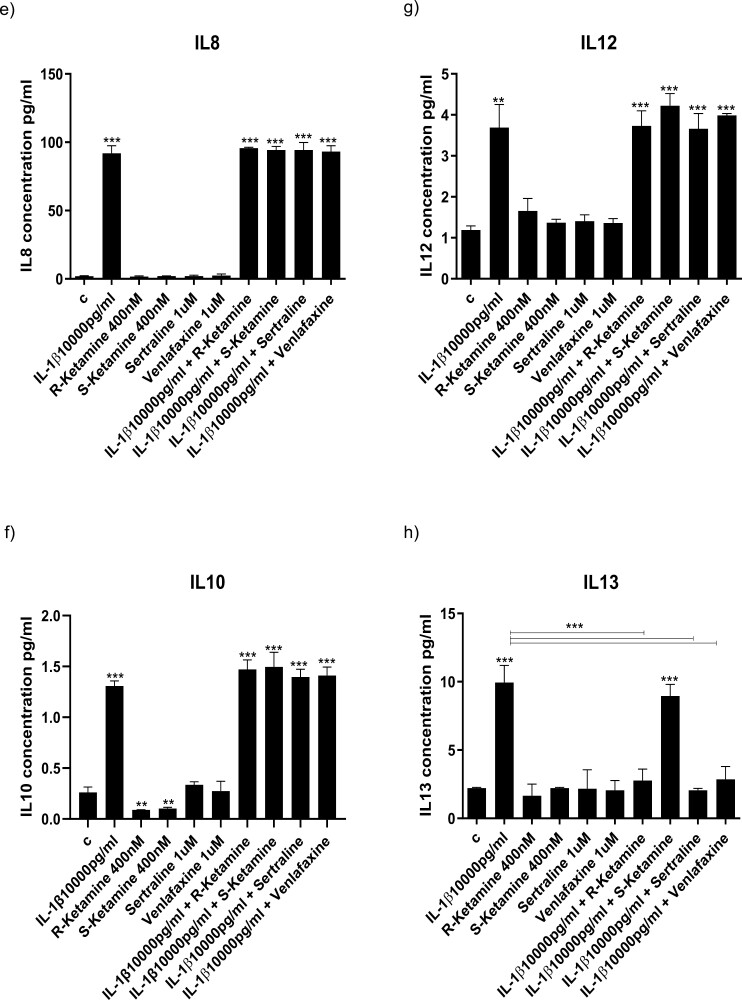

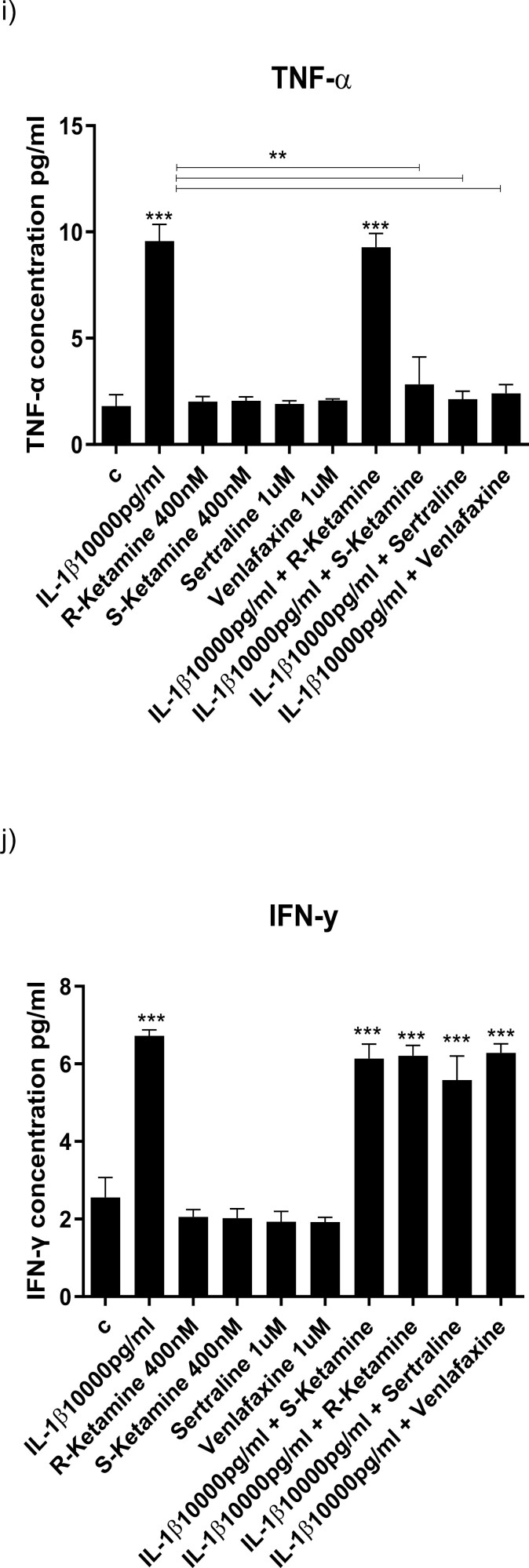
Cytokine release in the supernatant of cells exposed to IL-1b alone or in co-treatment with ketamine or antidepressants. Concentrations of cytokines in the supernatant of cells treated for 3 days during proliferation followed by 3 days during differentiation with IL-1b alone or in combination with R-ketamine, S-ketamine, sertraline, or venlafaxine. Treatment with IL-1b alone increased the concentration of all cytokines in the supernatant (A–J). Treatment with antidepressants alone increased the concentration of IL-2 (B) and decreased the concentration of IL-6 (D), while treatment with ketamine alone decreased the concentration of IL-4 (C), IL-6 (D), and IL-10 (F). R-ketamine, sertraline, and venlafaxine prevented the IL-1b–induced increase in IL-2 (B) and IL-13 (H). S-ketamine, sertraline, and venlafaxine prevented the IL-1b–induced increased production of TNF-a (I). Two-way ANOVA was performed. Data are shown as mean: *P < .05, **P < .01, ***P < .001 comparisons as indicated.

To establish the molecular mechanisms through which R-ketamine and S-ketamine prevents the anti-neurogenic effects of cytokines, we measured levels of cytokines in the supernatant after 3 days of differentiation. As we had previously shown (Borsini et al., 2021), treatment of cells with IL-1b increased the concentration of IL-1b, IL-2, IL-4, IL-6, IL-8, IL-10, IL-12, IL-13, TNF-a, and IFN-g compared with control (Figure 3A–J). Sertraline and venlafaxine had the broadest effects on cytokines, as they prevented the IL-1b–induced increased production of IL-2 (sertraline: from 5.5 to 1.5 pg/mL, *P < *.01; venlafaxine: from 5.5 1.5 pg/mL, *P < *.01; Figure 3B), IL-13 (sertraline: from 10 to 2 pg/mL, *P < *.001, venlafaxine: from 10 to 3 pg/mL, *P < *.001; Figure 3H), and TNF-a (sertraline: from 9 to 2 pg/mL, *P < *.01; venlafaxine: from 9 to 3 pg/mL, *P < *.01; Figure 3I). Co-treatment with R-ketamine and S-ketamine, instead, had more specific effects, albeit on the same cytokines: R-ketamine prevented the IL-1b–induced increased production of IL-2 (from 5.5 to 2 pg/mL, *P < *.01; Figure 3B) and IL-13 (from 10 to 3 pg/mL, *P < *.001; Figure 3H), while S-ketamine prevented the IL-1b–induced increased production of TNF-a (from 9 to 3 pg/mL, *P < *.01; Figure 3I).

**Figure 4.**
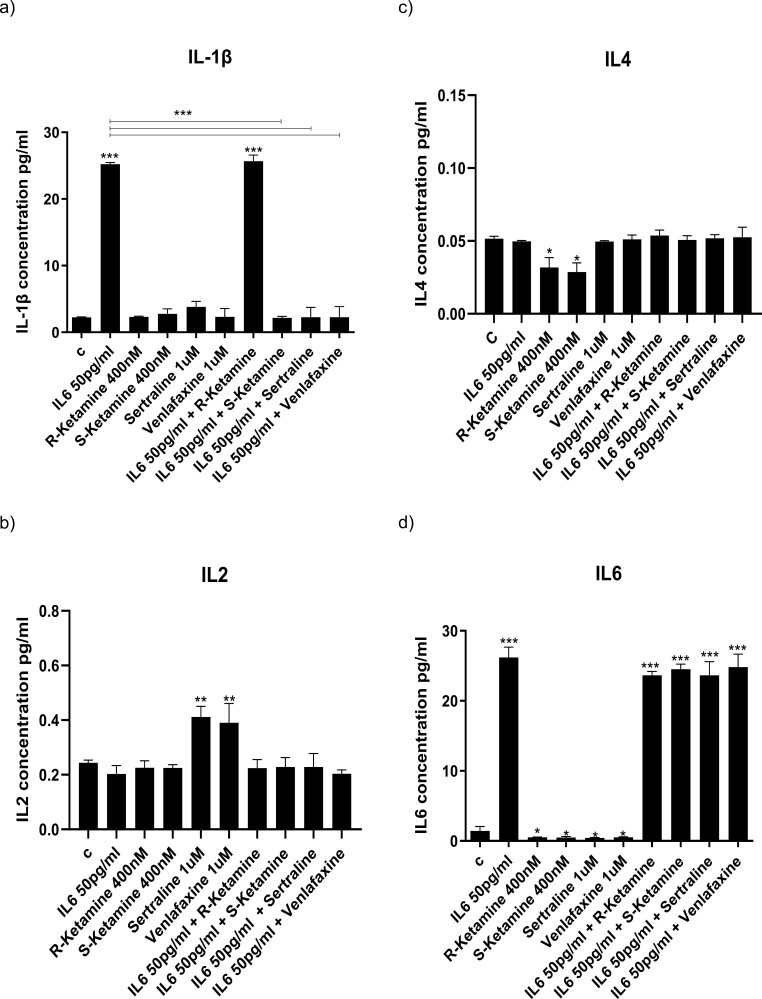

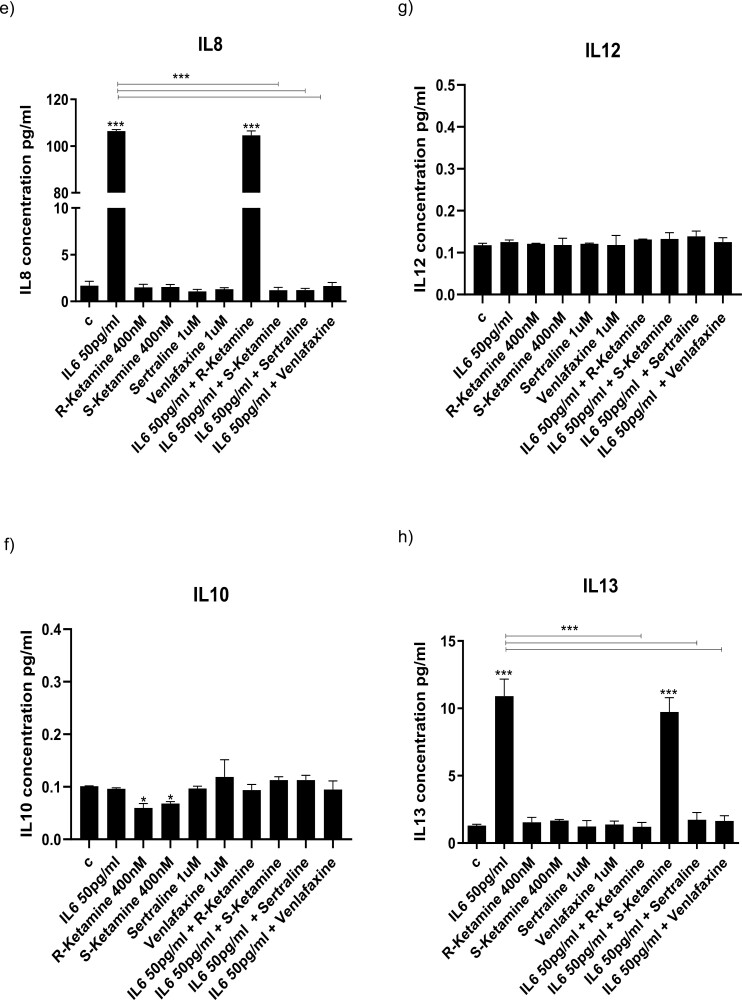

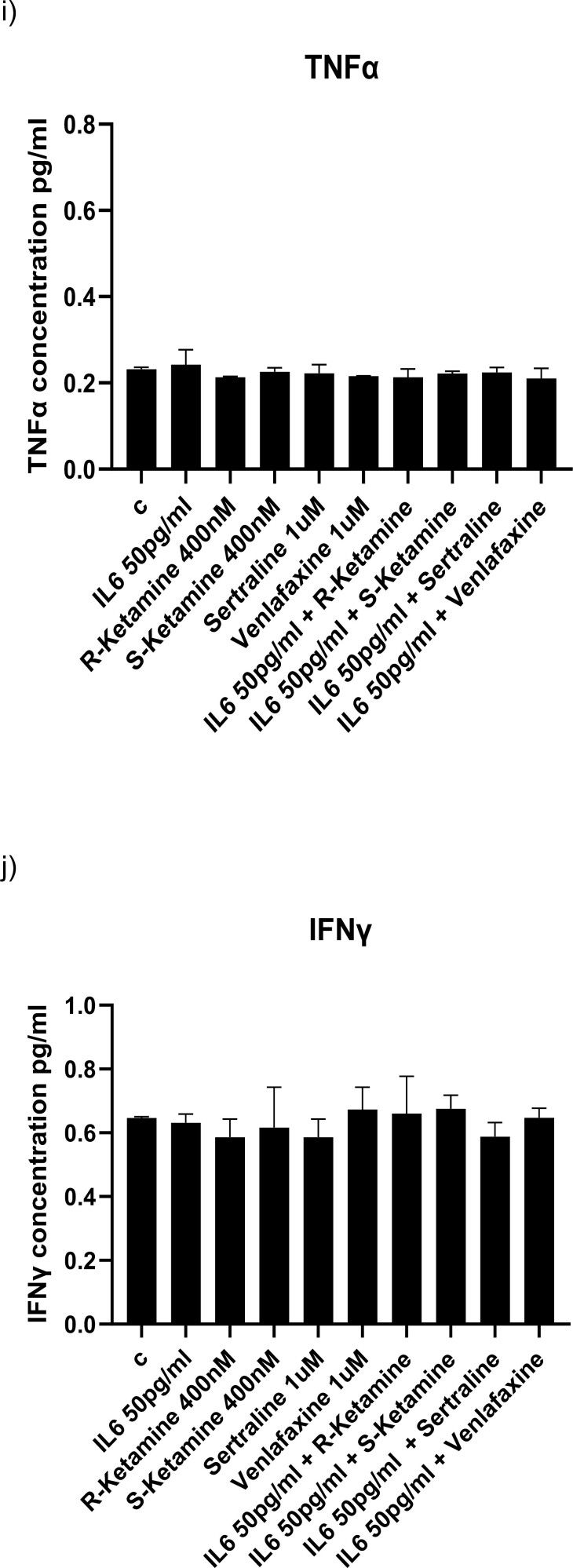
Production of cytokines in the supernatant of cells exposed to IL-6 alone or in co-treatment with ketamine or antidepressants. Concentrations of cytokines in the supernatant of cells treated for 3 days during proliferation followed by 3 days during differentiation with IL-6 alone or in combination with R-ketamine, S-ketamine, sertraline, or venlafaxine (A-J). Treatment with IL-6 alone increased the concentration of IL-1b (A), IL-6 (D), IL-8 (E), and IL-13 (H) in the supernatant. Treatment with antidepressants alone increased the concentration of IL-2 (B) and decreased the concentration of IL-6 (D), while treatment with ketamine alone decreased the concentration of IL-4 (C), IL-6 (D), and IL-10 (F). S-ketamine, sertraline, and venlafaxine prevented the IL-6–induced increase in IL-1b (A) and IL-8 (E). R-ketamine, sertraline, and venlafaxine prevented the IL-6–induced increased production of IL-13 (H). Two-way ANOVA was performed. Data are shown as mean: *P < .05, **P < .01, ***P < .001 comparisons as indicated.

A similar pattern was present with IL-6. Exposure of cells to IL-6 increased the concentration of IL-1b, IL-6, IL-8, and IL-13 (Figure 4A–J). Again, sertraline and venlafaxine had the broadest effects on cytokines, as they prevented IL-6–induced increased production of IL-1b (sertraline: from 25 to 2 pg/mL, *P < *.001, venlafaxine: from 25 to 2 pg/mL, *P < *.001; Figure 4A) and IL-8 (sertraline: from 110 to 2 pg/mL, *P < *.001, venlafaxine: from 110 to 2 pg/mL, *P < *.001; Figure 4E), and IL-13 (sertraline: from 12 to 1 pg/mL, *P < *.001, venlafaxine: from 12 to 1 pg/mL, *P < *.001; Figure 4H). Again, co-treatment with R-ketamine and S-ketamine had more specific effects: S-ketamine prevented the IL-6–induced increased production of IL-1b (from 25 to 2 pg/mL, *P < *.001; Figure 4A) and IL-8 (from 110 to 2 pg/mL, *P < *.001; Figure 4E), while co-treatment with R-ketamine prevented the IL-6–induced increased production of IL-13 (R-ketamine: from 12 to 1 pg/mL, *P < *.001; Figure 4H). These findings indicate that R-ketamine and S-ketamine modulate distinct inflammatory cytokines.

### Treatment With Antibodies against IL-2, IL-13, and TNF-a, and IL-1b, IL-8, and IL-13 Prevents, Respectively, IL-1b– and IL-6–Induced Reduction of Neurogenesis and Increase of Apoptosis

**Figure 5.**
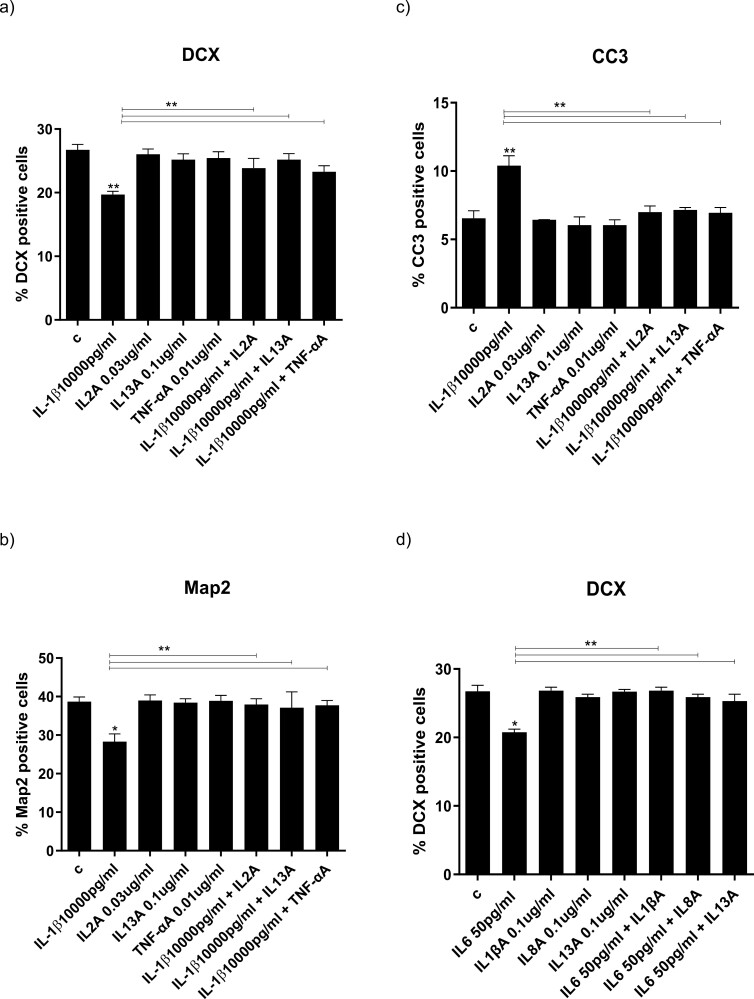

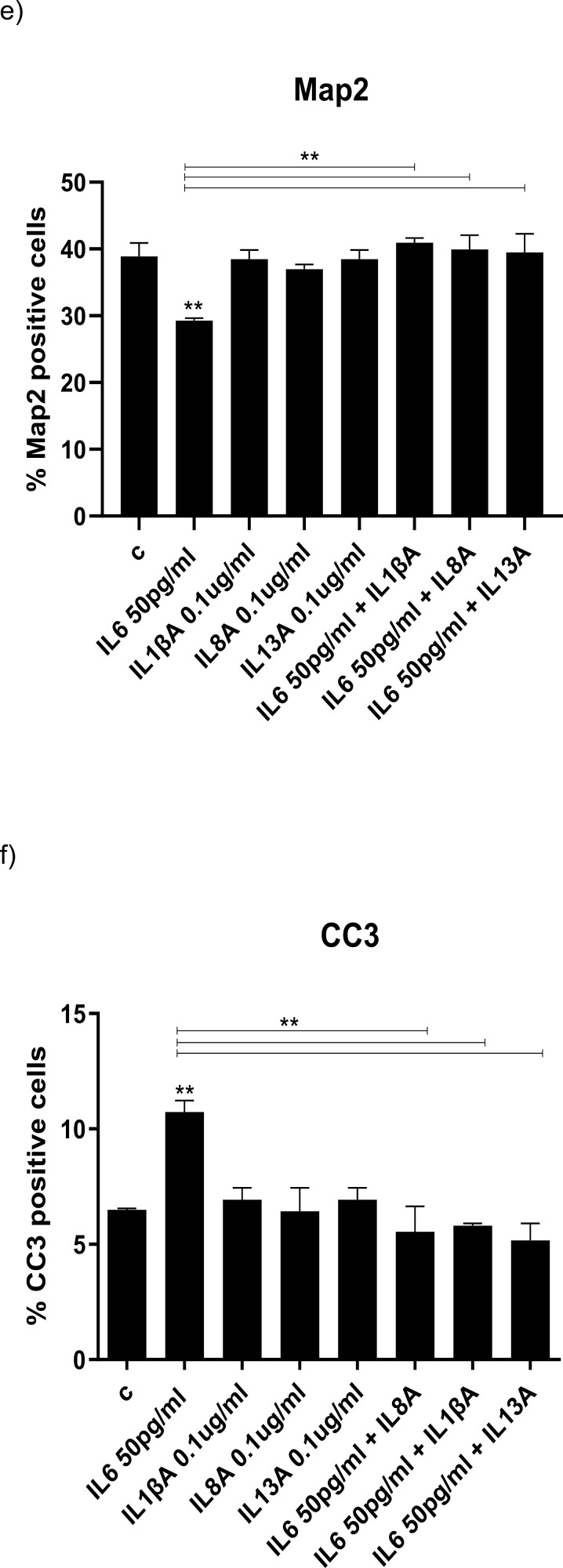
Antibodies against IL-2, IL-13, and TNF-a, and IL-1b, IL-8 and IL-13 prevent, respectively, IL-1b– and IL-6–induced reductions in neurogenesis and increases in apoptosis. Treatment with IL-2A (0.03 mg/mL), IL-13A (0.1 mg/mL), and TNF-aA (0.01 mg/mL) prevents the decrease in DCX- and MAP2-positive cells and the increase in CC3-positive cells induced by IL-1b (A–C). Treatment with IL-1bA (0.1 mg/mL), IL-8A (0.1 µg/mL), and IL-13A (0.1 mg/mL) prevents the decrease in DCX- and MAP2-positive cells and the increase in CC3-positive cells induced by IL-6 (D–F). Two-way ANOVA was performed. Data are shown as mean; *P < .05, **P < .01 comparisons as indicated.

To confirm that the previously identified cytokines were indeed responsible for the beneficial effects exerted by each ketamine enantiomer and antidepressants in the presence of either IL-1b (through IL-2, IL-13, and TNF-a) or IL-6 (through IL-1b, IL-8, and IL-13), we treated cells with IL-1b or IL-6, but in this case we added antibodies against the specific downstream cytokines. Similar to treatment with either ketamine enantiomer or antidepressants, treatment of cells with either anti-IL-2 antibody (IL-2A) (0.03 µg/mL), IL-13A (0.1 µg/mL), or TNF-aA (0.01 µg/mL) prevented the IL-1b–induced decrease in DCX-positive (IL-2A: +7%, *P < *.01 vs IL-1b, IL-13A: +6%, *P < *.01 vs IL-1b, TNF-aA: +7%, *P < *.01 vs IL-1b; Figure 5A) and MAP2-positive cells (IL-2A: +10%, *P < *.01 vs IL-1b, IL-13A: +9%, *P < *.01 vs IL-1b, TNF-aA: +1%, *P < *.01 vs IL-1b; Figure 5B) and increase in CC3-positive cells (IL-2A: −5%, *P < *.01 vs IL-1b, IL-13A: −6%, *P < *.01 vs IL-1b, TNF-aA: −6%, *P < *.01 vs IL-1b; Figure 5C). Again, similar to treatment with either ketamine enantiomer or antidepressants, treatment with either IL-1bA (0.1 µg/mL), IL-8A (0.1 µg/mL), or IL-13 A (0.1 µg/mL) prevented the IL-6–induced reduction of DCX-positive cells (IL-1bA: +6%, *P < *.01 vs IL-6, IL-8A: +5%, *P < *.01 vs IL-6, IL-13A: +5%, *P < *.01 vs IL-6; Figure 5D) and MAP2-positive cells (IL-1bA: +10%, *P < *.01 vs IL-6, IL-8A: +9%, *P < *.01 vs IL-6, IL-13A: +10%, *P < *.01 vs IL-6; Figure 5E), as well as increase of CC3-positive cells (IL-1bA: −5%, *P < *.01 vs IL-6, IL-8A: −7%, *P < *.01 vs IL-6, IL-13A: −5%, *P < *.01 vs IL-6; Figure 5F). Therefore, these findings confirm the involvement of these cytokines as a mechanism behind the ability of R-ketamine, S-ketamine, and antidepressants to reverse cytokine-induced reductions of neurogenesis and increase apoptosis.

### Ketamine Enantiomers and Antidepressants Prevent IL-1b– but Not IL-6–Induced Activation of Kynurenine Pathway via Inhibiting IL-2, IL-13 and TNFa

Having shown the ability for ketamine enantiomers and antidepressants to inhibit the IL-1b– and IL-6–induced production of downstream cytokines, which are responsible for the detrimental effects observed on neurogenesis and apoptosis, we subsequently measured candidate metabolites of the kynurenine pathway, again in the presence of IL-1b or IL-6, either alone or in co-treatment with R-ketamine, S-ketamine, or antidepressants, in supernatant of cells after 3 days of differentiation.

**Figure 6.**
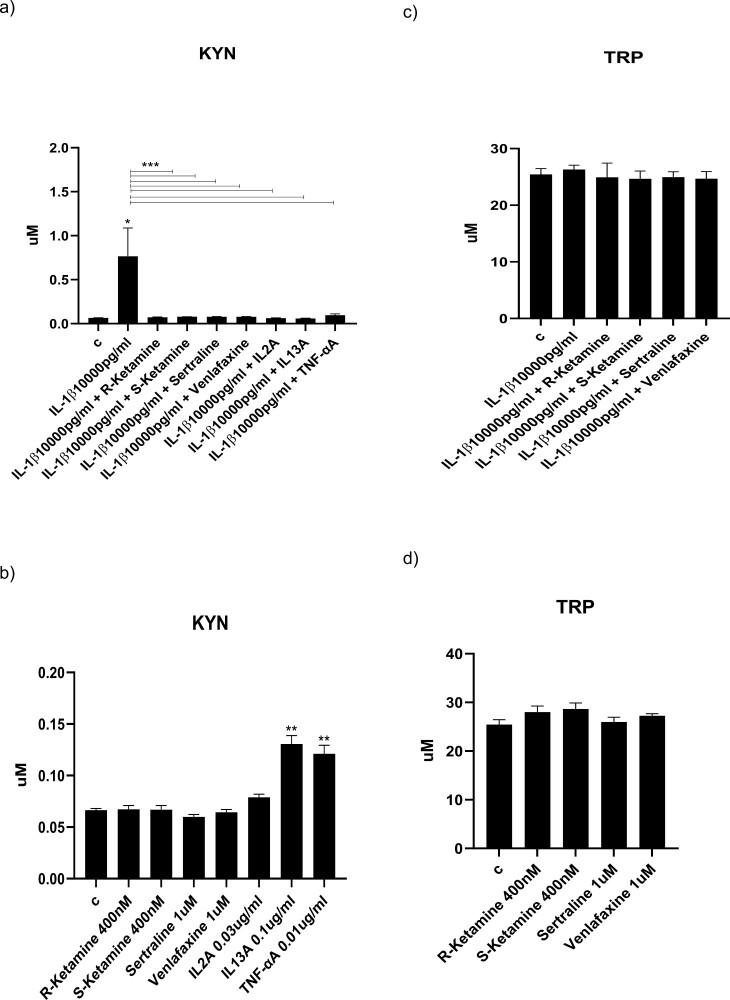

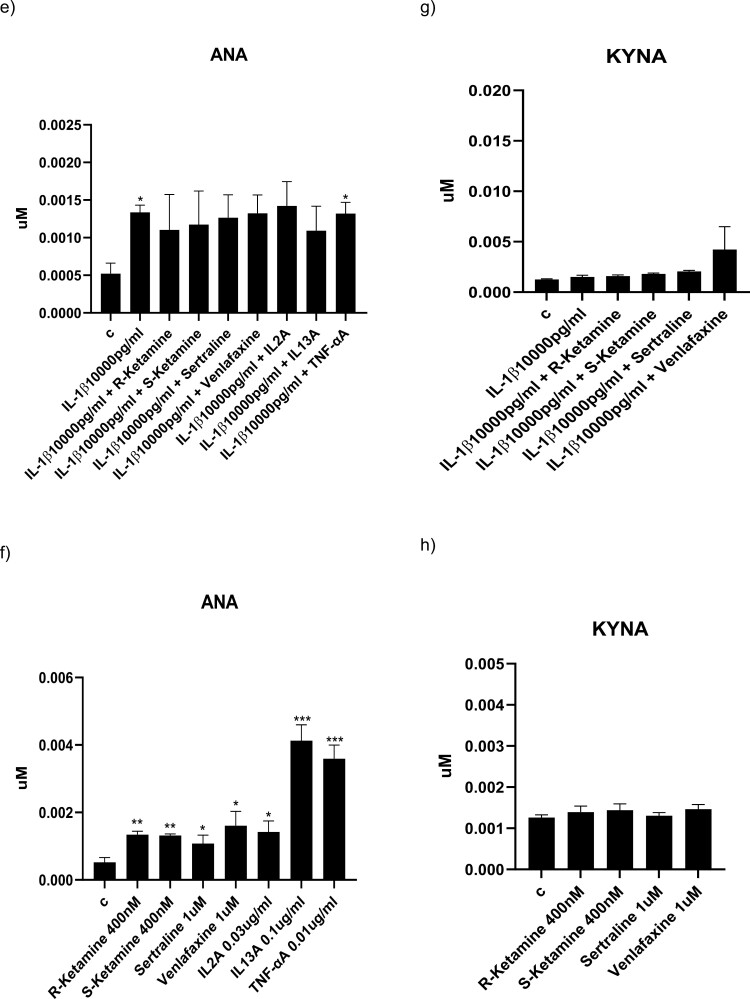

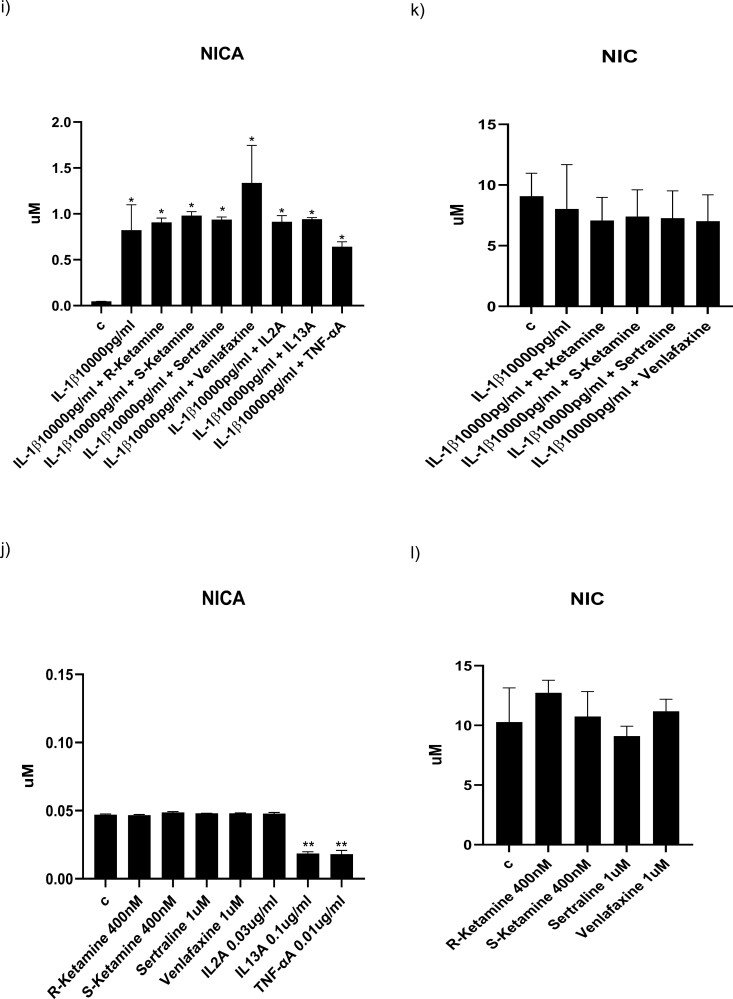

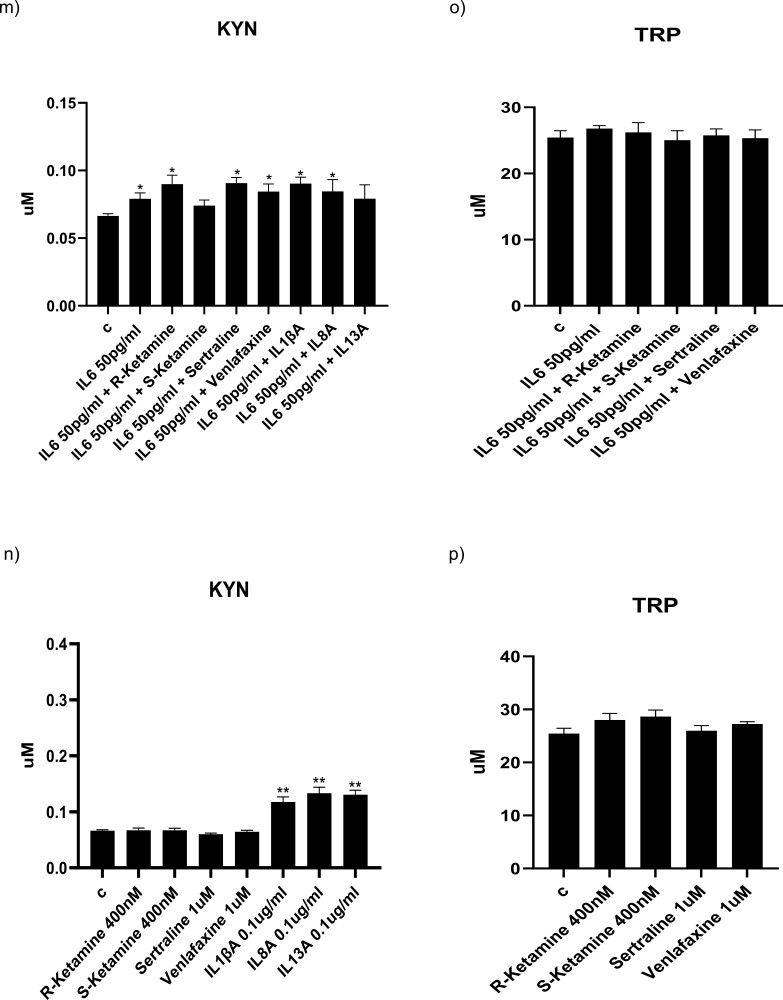

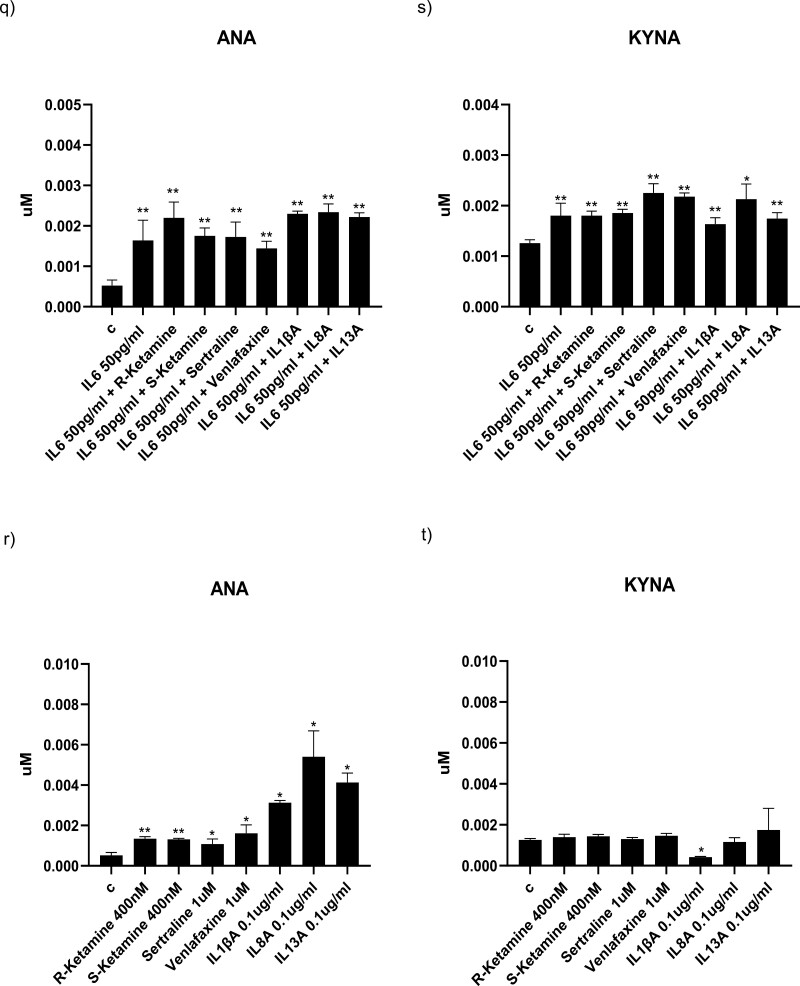

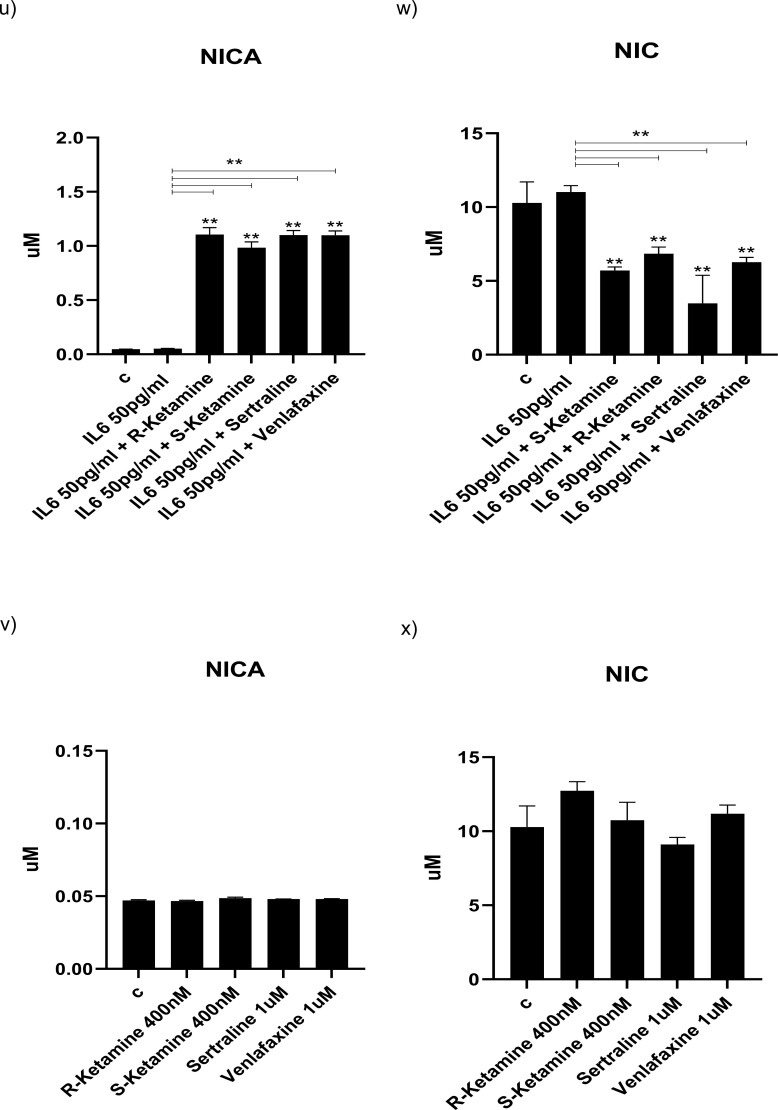
Ketamine and antidepressants prevent IL-1b– but not IL-6–induced activation of the kynurenine pathway via inhibiting IL-2, IL-13, and TNFα. Concentrations of kynurenine pathway metabolites in the supernatant of cells treated for 3 days during proliferation followed by 3 days during differentiation with IL-1b or IL-6 alone or in combination with R-ketamine, S-ketamine, sertraline, or venlafaxine (A–X). Treatment with IL-1b alone increased the concentration of kynurenine (KYN) (A), anthranilic acid (E), and nicotinic acid (NICA) (I). Co-treatment with R-ketamine, S-ketamine, sertraline, venlafaxine, IL-2A, IL-13A, or TNF-aA prevents the IL-1b–induced increased production of KYN (A). Treatment with R-ketamine, S-ketamine, sertraline, venlafaxine, IL-2A, IL-13A, or TNF-a alone increases the concentration of anthranilic acid (F). Treatment with IL-13A or TNF-a alone increases the concentration of KYN (A) and decreases the concentration of NICA (J). Treatment with IL-6 increased production of KYN (M), ANA (Q), and KYNA (S), none of which were prevented by co-treatment with R-ketamine, S-Ketamine, sertraline, venlafaxine, IL-1bA, IL-8A, or IL-13A. Treatment with IL-1bA, IL-8A, or IL-13A alone increased the concentration of KYN (N), while R-ketamine, S-ketamine, sertraline, venlafaxine, IL-1bA, IL-8A, or IL-13A alone increased the concentration of ANA (R) and NICA (U) and decreased the concentration of nicotinamide (NIC) (W). Two-way ANOVA (A, C, E, G, I, K, M, O, Q, S, U, W) and 2-way ANOVA (B, D, F, H, J, L, N, P, R, T, V, X) were performed. Data are shown as mean: *P < .05, **P < .01, ***P < .001 comparisons as indicated.

In particular, IL-1b induced the production of KYN (+1 µM, *P < *.001, vs vehicle), ANA (+0.001 µM, *P < *.05, vs vehicle), and NICA (+1 µM, *P < *.05, vs vehicle), while no changes were observed for TRP, KYNA, or NIC (Figure 6A–L). Interestingly, co-treatment with either R-ketamine, S-ketamine, sertraline, or venlafaxine fully prevented the IL-1b–induced increased concentration of KYN (Figure 6A) but not of the other 2 metabolites (Figure 6A, E, I). Similar effects to ketamine enantiomers and antidepressants were observed when cells were treated with IL-1b and IL-2A, IL-13A, or TNFaA (Figure 6A, E, I). This suggests that the production of KYN is indeed mediated by (IL-1b–induced) IL-2, IL-13, and TNFa and that both ketamine enantiomers and antidepressants are able to inhibit the production of KYN via acting on the same cytokines. The concentration of 3-HK, 3-HANA, and QUIN were below the detection limit for all treatment conditions (data not shown).

Similarly, IL-6 induced the production of KYN (+0.02 µM, *P < *.05, vs vehicle), ANA (+0.001 µM, *P < *.01, vs vehicle), and, differently from IL-1b, of the neuroprotective KYNA (+0.0009 µM, *P < *.01, vs vehicle), while no changes were observed for TRP, NICA, or NIC (Figure 6M–X). Interestingly, in this case, co-treatment with R-ketamine, S-ketamine, sertraline, or venlafaxine did not prevent IL-6–induced increased concentration of any of these metabolites (Figure 6M, Q, S). Similarly, treatment of cells with IL-6 and either IL-1bA, IL-8A, or IL-13A (Figure 6M, Q, S) also did not prevent IL-6–induced increased concentration of any of these metabolites. This suggests that the production of KYN, ANA, and KYNA is not mediated by (IL-6–induced) IL-1bA, IL-8A, or IL-13A, which are instead a target of R-ketamine, S-ketamine, and antidepressants. Again, the concentrations of 3-HK, 3-HANA, and QUIN were below the detection limit for all treatment conditions (data not shown).

**Figure 7.**
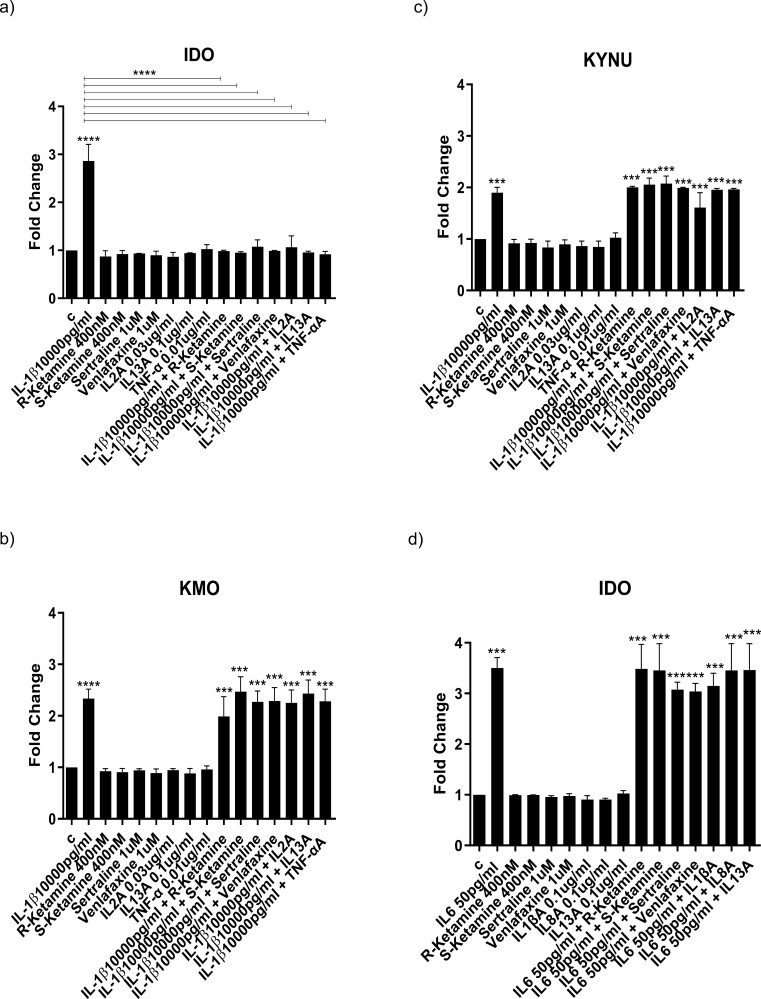

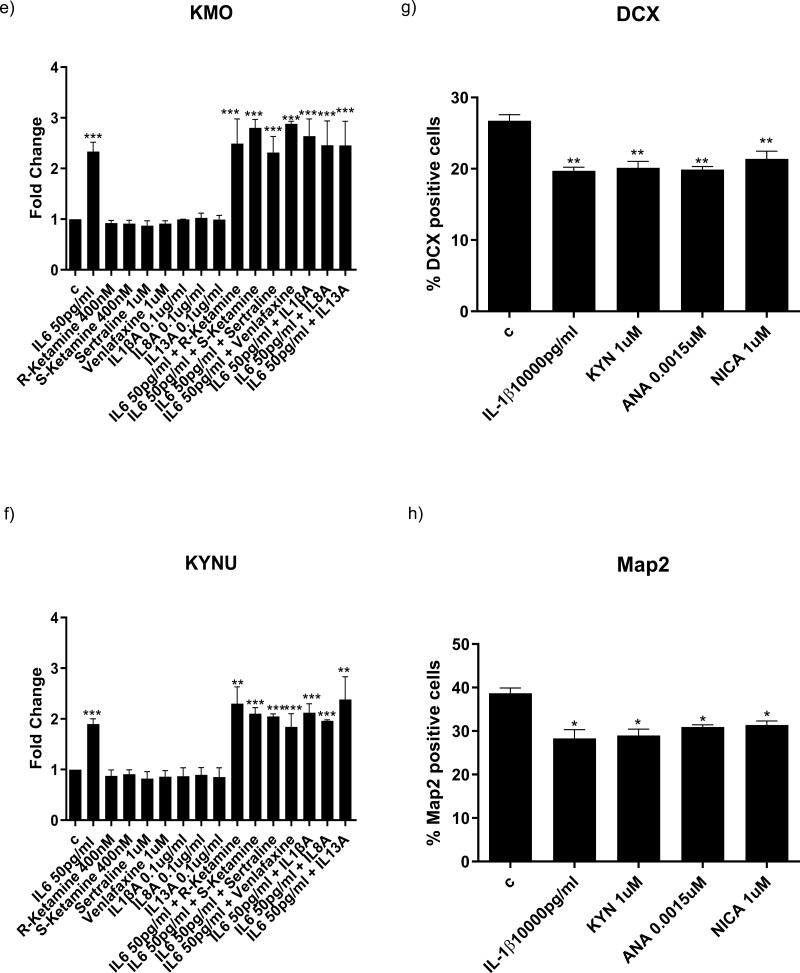

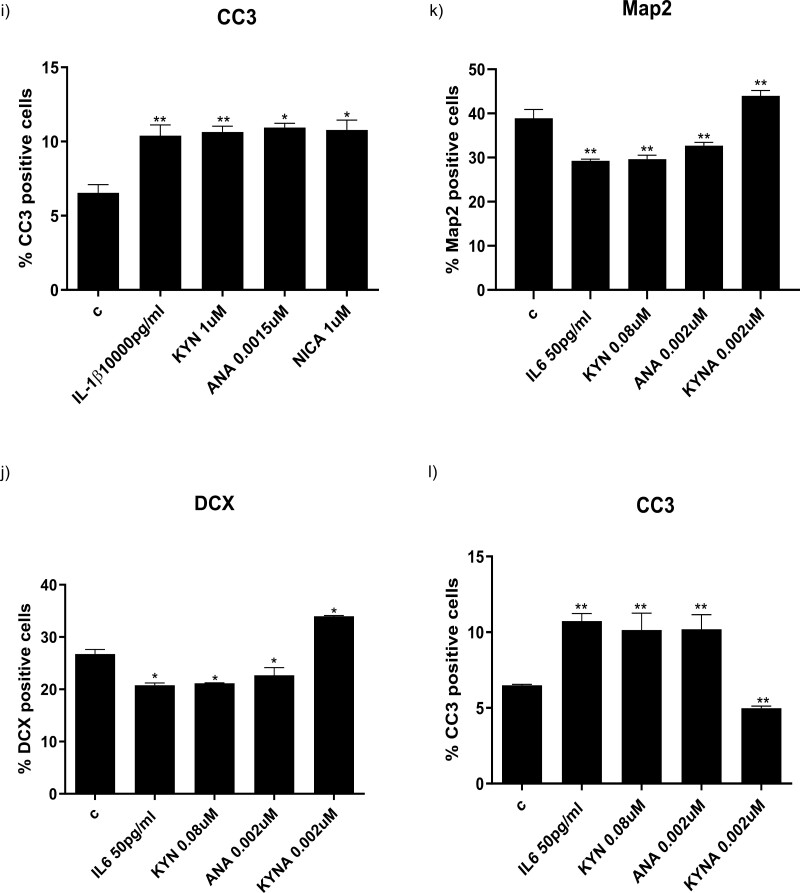
Expression of genes involved in the activation of the kynurenine pathway, and the effect on cells of IL-1b– and IL-6–induced kynurenine metabolites. Gene expression changes in cells treated for 3 days during proliferation followed by 2 days during differentiation with IL-1b or IL-6 alone or in combination with R-ketamine, S-ketamine, sertraline, or venlafaxine. Treatment with IL-1b increased expression of IDO, KMO, and KYNU (A–C). Co-treatment with R-ketamine, S-ketamine, sertraline, venlafaxine, and IL-2A, IL-13A, and TNF-aA prevented the IL-1b–induced increased expression of IDO (A). Treatment with IL-6 alone increased the concentrations of IDO, KMO, and KYNU (D–F); however, none of these increases were prevented by co-treatment with R-ketamine, S-ketamine, sertraline, venlafaxine, or antibodies for IL-1b, IL-8, and IL-13. Additionally, cells were treated between day 3 and 7 of differentiation directly to the same concentrations of KYN, ANA, NICA, or KYNA previously identified upon treatment with IL-1b or IL-6 alone. Treatment of cells with KYN (1 mM), ANA (0.0015 mM), and NICA (1 mM) decreased the percentage of DCX- and MAP2-positive neurons and increased the percentage of CC3-positive neurons to the same degree as IL-1b treatment (G–I). Similarly, KYN (0.08 mM) and ANA (0.002 mM) decreased the percentage of DCX- and MAP2-positive neurons and increased the percentage of CC3-positive neurons to the same degree as IL-6 treatment (J–L). Conversely, treatment with KYNA (0.002 mM) increased the percentage of DCX- and MAP2-positive neurons and decreased the percentage of CC3-positive neurons compared with control (J–L). Two-way ANOVA (A–F), and 2-way ANOVA (G–L) were performed. Data are shown as mean; *P < .05, **P < .01, ***P < .001, ****P < .0001 comparisons as indicated.

We confirmed activation of the kynurenine pathway by measuring the expression of the enzymes IDO, KMO, and KYNU upon treatment with IL-1b (IDO: +200%, *P < *.0001 vs vehicle, KMO: +150%, *P < *.0001 vs vehicle, KYNU: +100%, *P < *.001 vs vehicle; Figure 7A–C) and IL-6 (IDO: +250%, *P < *.001 vs vehicle, KMO: +150%, *P < *.001 vs vehicle, KYNU: +100%, *P < *.001 vs vehicle; Figure 7D–F) after 2 days during differentiation. Interestingly, and in line with previous findings, co-treatment with ketamine enantiomers, antidepressants, IL-2A, IL-13A, or TNFaA fully prevented the IL-1b–induced upregulation of IDO expression but not of KMO and KYNU (Figure 7A–C). However, co-treatment with R-ketamine, S-ketamine antidepressants, IL-1bA, IL-8A, or IL-13A did not prevent the IL-6–induced increase in IDO, KMO, and KYNU (Figure 7D–F). Again, this is in line with previous findings from this study whereby ketamine enantiomers and antidepressants are able to inhibit kynurenine pathway activation only in presence of IL-1b but not of IL-6.

### Treatment of Cells With IL-1b- or IL-6-Induced Kynurenine Metabolites Decreases Neurogenesis and Increases Apoptosis

To test whether these identified kynurenine metabolites were able to have detrimental effects on neurogenesis and apoptosis, we exposed cells (between day 3 and 7 of differentiation) directly to the same concentrations of KYN, ANA, NICA, or KYNA previously identified upon treatment with IL-1b or IL-6 alone. Results showed that KYN (1 mM), ANA (0.0015 mM), and NICA (1 mM) decreased the percentage of DCX-positive cells (KYN: −7% vs vehicle, *P < *.01, ANA: −7% vs vehicle, *P < *.01, NICA: −7% vs vehicle, *P < *.01; Figure 7G) and MAP2-positive cells (KYN: −11% vs vehicle, *P < *.05, ANA: −10% vs vehicle, *P < *.05, NICA: −10% vs vehicle, *P < *.01; Figure 7H) and increased the percentage of CC3-positive cells (KYN: +4% vs vehicle, *P < *.01, ANA: +5% vs vehicle, *P < *.05, NICA: 5% vs vehicle, *P < *.05; Figure 7I) to the same level as treatment with IL-1b. Additionally, treatment with KYN (0.08 mM) and ANA (0.002 mM) decreased the percentage of DCX-positive cells (KYN: −8% vs vehicle, *P < *.05, ANA: −7% vs vehicle, *P < *.05; Figure 7J) and MAP2-positive cells (KYN: −10% vs vehicle, *P < *.01, ANA: −8% vs vehicle, *P < *.01; Figure 7K) and increased the percentage of CC3-positive cells (KYN: +5% vs vehicle, *P < *.01, ANA: +5% vs vehicle, *P < *.01; Figure 7L) to the same level as treatment with IL-6. Conversely, treatment of cells with (the neuroprotective) KYNA at concentrations found in the supernatant of cells treated with IL-6 (0.002 mM) increased the percentage of DCX-positive cells (KYNA: +4% vs vehicle, *P < *.05; Figure 7J) and MAP2-positive cells (KYNA: +5% vs vehicle, *P < *.01; Figure 7K) and decreased the percentage of CC3-positive cells (KYNA: −2% vs vehicle, *P < *.01; Figure 7L) compared with IL-6 treatment. Overall, these results suggest that R-ketamine and S-ketamine prevent the anti-neurogenic effects of IL-1b via inhibition of cytokine production and subsequent inhibition of the cytokine-activated kynurenine pathway. However, for IL-6, R-ketamine and S-ketamine prevent the anti-neurogenic effects via inhibition of cytokine production but *not* via subsequent inhibition of the cytokine-activated kynurenine pathway.

## DISCUSSION

Our results show, to our knowledge for the first time, that treatment of HPCs with both R- and S-ketamine prevents IL-1b– and IL-6–induced reductions in hippocampal neurogenesis in vitro by inhibiting cytokine production downstream of IL-1b and IL-6. Interestingly, both R- and S-ketamine inhibit the (neurotoxic) kynurenine pathway only in response to IL-1b. Additionally, to the best of our knowledge, this is the first study to directly compare the neuroprotective effects of the 2 ketamine enantiomers, S-ketamine and R-ketamine, and we demonstrate that they have distinct immunomodulatory profiles.

Treatment of cells with R-ketamine or S-ketamine is equally efficacious in preventing the IL-1b– and IL-6–induced reductions in neurogenesis. The efficacy of ketamine specifically in preventing cytokine-induced reductions in neurogenesis is of particular relevance given the role that the immune system plays in depression, particularly in treatment-resistant patients (Strawbridge et al., 2015; Cattaneo et al., 2020b). Indeed, patients with treatment-resistant depression and a higher inflammatory milieu have a better response to anti-inflammatory medications, including ketamine (Yang et al., 2019; Nettis et al., 2021). Additionally, we demonstrate that both ketamine enantiomers (400 nM) prevent the increase in apoptosis induced by IL-6 and IL-1b. This is in alignment with previous studies in cultured rodent NPCs, whereby treatment with similar concentrations of racemic ketamine, containing both R- and S-enantiomers, promotes neuronal survival and attenuates apoptosis (Wu et al., 2023; Zhao et al., 2023).

Our results demonstrate that treatment of HPCs with IL-1β or IL-6 increases levels of cytokines, as established in our previous studies (Borsini et al., 2020a, 2021, 2022). However, co-treatment with R-ketamine or S-ketamine attenuates this increase. Specifically, the ketamine enantiomers prevent IL-1β–induced elevations in IL-2, IL-13, and TNF-α, as well as IL-6–induced elevations in IL-1β, IL-8, and IL-13. These findings are in accordance with clinical and preclinical studies, whereby ketamine treatment reduces circulating levels of cytokines alongside increasing neurogenesis (Dong et al., 2012; Yang et al., 2013a, 2013b; Clarke et al., 2017; Zhou et al., 2020; Kopra et al., 2021; Sukhram et al., 2022). Furthermore, cytokine reductions are most prevalent in patients who respond to ketamine, highlighting the role of this decrease in alleviating depressive symptoms (Yang et al., 2015b; Nikkheslat, 2021).

Of note, our findings reveal that the 2 ketamine enantiomers exhibit distinct immunomodulatory properties. Specifically, R-ketamine attenuates IL-1b–induced IL-2 and IL-13, as well as IL-6–induced IL-13. In contrast, S-ketamine mitigates IL-1b–induced TNF-a, as well as IL-6–induced IL-1b and IL-8, highlighting the importance of considering ketamine formulation in depression treatment. Also, the addition of an antibody to any of these cytokines inhibited by either ketamine enantiomer prevents the detrimental effects of IL-1b and IL-6 to the same degree as R-ketamine or S-ketamine treatment alone, highlighting their role in mediating the anti-neurogenic effects of IL-1b and IL-6. Indeed, higher IL-13 levels are associated with more severe depression and a higher number of suicide attempts (Vai et al., 2021), and elevated levels of IL-2 (Suhee et al., 2023), TNF-a (Tuglu et al., 2003), and IL-8 (Kuzior et al., 2020) are associated with depressive symptoms in clinical studies. Certainly, a recent meta-analysis concluded that the racemic ketamine formulation, which contains both R-ketamine and S-ketamine, is a more effective antidepressant than S-ketamine alone (Nikolin et al., 2023), indicating that R-ketamine also has, or participates in, the antidepressant effects. Indeed, while not tested clinically, preclinical studies have shown R-ketamine be a more efficacious antidepressant than S-ketamine (Fukumoto et al., 2017; Chang et al., 2019). Considering the present study, it is tempting to speculate that the clinical superiority of the racemic formulation may stem from the synergistic action of both enantiomers in mitigating production of a broader spectrum of cytokines. Even though our results indicate both enantiomers are equally efficacious in alleviating anti-neurogenic effects, their divergent anti-inflammatory profile highlights the possibility of stratifying patients to a ketamine formulation depending on their distinct inflammatory milieu.

The role of the kynurenine pathway in inflammation-induced depression has become increasingly apparent (Borsini et al., 2017; Savitz, 2020; Kopra et al., 2021), and studies have highlighted the ability of ketamine to modulate this pathway (Kopra et al., 2021). The results presented here and previous findings from our group have established that IL-1b treatment modulates the kynurenine pathway in vitro, with increased activity of the neurotoxic arm associated with decreased neurogenesis (Zunszain et al., 2012; Borsini et al., 2017). Therefore, we wanted to explore if ketamine’s ability to prevent the anti-neurogenic effects of cytokines may be mediated via modulation of the kynurenine pathway. Indeed, we find that the IL-1b–induced increase in both the concentration of kynurenine and the expression of IDO can be reversed via co-treatment with either R-ketamine and S-ketamine or with antibodies for IL-2, IL-13, or TNF-a. Therefore, the capacity of the ketamine enantiomers to rescue the anti-neurogenic effects of IL-1b is underscored by its ability to inhibit IL-1b–induced IL-2, IL-13, and TNF-a and subsequent production of KYN. Indeed, we also demonstrated that treatment of cells with KYN at the concentration induced by IL-1b (1 mM) is sufficient to decrease neurogenesis, highlighting that KYN is responsible for this decrease. While previous preclinical and clinical studies have found correlations between cytokines, ketamine, and kynurenine at a systemic level, this study is the first, to our knowledge, to reveal clear mechanistic, causal pathways (Kadriu et al., 2021; Kopra et al., 2021).

It is noteworthy that in this study, co-treatment with R-ketamine or S-ketamine and IL-6 influenced the kynurenine pathway distinctly compared with IL-1b in that none of the kynurenine pathway changes induced by IL-6 were influenced by treatment with either ketamine enantiomer or antibodies for IL-1b, IL-13, or IL-8. This suggests that these cytokines are not the mediators of IL-6–induced changes in kynurenine pathway metabolites. Rather, it may simply be that IL-6 directly modulates the kynurenine pathway independently from the induction of downstream cytokine production (Kim et al., 2012).

Moreover, we also revealed that IL-6 treatment increases production not only of the neurotoxic KYN and ANA but also of the neuroprotective KYNA (Savitz et al., 2015). Indeed, we also found that KYNA alone promotes neurogenesis above the control group. Therefore, despite IL-6’s potential to enhance some neurotoxic kynurenine metabolites, the concurrent production of KYNA may mitigate their detrimental effects on neurogenesis. This suggests that the kynurenine pathway is not the sole mechanism underlying the anti-neurogenic effects of IL-6 and thus raises speculation that the protective effects of the ketamine enantiomers in this context involve another pathway downstream of IL-6–induced IL-1b, IL-13, or IL-8. For example, in a previous study from our group, we demonstrated that the detrimental effects of IL-6–induced IL-13 production on neural progenitor cell differentiation could be prevented via treatment with Janus Kinase inhibitors (Borsini et al., 2022).

While this study was robust in its design, we acknowledge there are potential limitations. First, we used an in vitro model of hippocampal neurogenesis with an immortalized cell line. While this does not fully recapitulate the adult neurogenic niche, we previously replicated findings from this model in both animal and clinical studies, including changes in neurogenesis by cortisol, cytokines, and antidepressants (Anacker et al., 2013a, 2013b; Hack et al., 2016; Hepgul et al., 2016; Cattaneo et al., 2018, 2020a; Cattane et al., 2019; Borsini et al., 2020a, 2021, 2022; Horowitz et al., 2020). Based on this, we are confident that our results translate to the human brain. Additionally, while the cells used here do possess capabilities to differentiate into astrocytes, we did not assess how cytokines or ketamine influence astrogliogenesis. In future studies, we aim to extend these findings and explore whether ketamine may mitigate glia-related adaptations, which have been shown in other in vitro models to be induced by cytokine challenges (Benson et al., 2020).

In summary, our study reveals novel mechanistic information on both R- and S-ketamine’s ability to prevent the detrimental effects of inflammation on neurogenesis, a putative pathway underpinning the pathogenesis of (inflammation-induced) depression. We demonstrate that both R- and S-ketamine are equally efficacious in preventing the anti-neurogenic effects of cytokines, although they have different anti-inflammatory profiles. Further, both R- and S-ketamine appear to exert protective effects on neurogenesis via modulation of the kynurenine pathway but only in protection against the detrimental effects of IL-1b, not IL-6. Further studies are required to enhance our understanding of the mechanisms of ketamine’s antidepressant effects to provide more effective personalized therapeutic approaches to depression.

## Supplementary Material

pyae041_suppl_Supplementary_Figure_S1

pyae041_suppl_Supplementary_Figure_S2

## Data Availability

Data that support the present results are available from the corresponding author upon reasonable request.

## Interest Statement

Dr Alessandra Borsini and Professor Carmine M. Pariante have received research funding also from Johnson & Johnson for research on depression and inflammation which included cellular work (2012–2018), but this work is unrelated to that funding; moreover, less than 10% of Professor Pariante’s support in the last 10 years derives from commercial collaborations, including: a strategic award from the Wellcome Trust (Neuroimmunology of Mood Disorders and Alzheimer’s Disease [NIMA] Consortium, grant 104025), in partnership with Janssen, GlaxoSmithKline, Lundbeck, and Pfizer; and consultation and speakers fees from Boehringer Ingelheim, Eli Lilly, Compass, Eleusis, GH Research, Lundbeck, and Värde Partners. Drs Borsini, Alboni, Mandal, and Kirkpatrick have no conflicts of interest to declare.

## References

[CIT0001] Anacker C , Cattaneo A, Luoni A, Musaelyan K, Zunszain PA, Milanesi E, Rybka J, Berry A, Cirulli F, Thuret S, Price J, Riva MA, Gennarelli M, Pariante CM (2013a) Glucocorticoid-related molecular signaling pathways regulating hippocampal neurogenesis. Neuropsychopharmacology 38:872–883. 23303060 10.1038/npp.2012.253 PMC3672002

[CIT0002] Anacker C , Cattaneo A, Musaelyan K, Zunszain PA, Horowitz M, Molteni R, Luoni A, Calabrese F, Tansey K, Gennarelli M, Thuret S, Price J, Uher R, Riva MA, Pariante CM (2013b) Role for the kinase SGK1 in stress, depression, and glucocorticoid effects on hippocampal neurogenesis. Proc Natl Acad Sci USA 110:8708–8713. 23650397 10.1073/pnas.1300886110 PMC3666742

[CIT0003] Autry AE , Adachi M, Nosyreva E, Na ES, Los MF, Cheng PF, Kavalali ET, Monteggia LM (2011) NMDA receptor blockade at rest triggers rapid behavioural antidepressant responses. Nature 475:91–95. 21677641 10.1038/nature10130 PMC3172695

[CIT0004] Beilin B , Rusabrov Y, Shapira Y, Roytblat L, Greemberg L, Yardeni IZ, Bessler H (2007) Low-dose ketamine affects immune responses in humans during the early postoperative period. Br J Anaesth 99:522–527. 17681970 10.1093/bja/aem218

[CIT0005] Benson CA , Powell HR, Liput M, Dinham S, Freedman DA, Ignatowski TA, Stachowiak EK, Stachowiak MK (2020) Immune factor, TNFα, disrupts human brain organoid development similar to schizophrenia-schizophrenia increases developmental vulnerability to TNFα. Front Cell Neurosci 14:233. 33005129 10.3389/fncel.2020.00233 PMC7484483

[CIT0006] Berman RM , Cappiello A, Anand A, Oren DA, Heninger GR, Charney DS, Krystal JH (2000) Antidepressant effects of ketamine in depressed patients. Biol Psychiatry 47:351–354. 10686270 10.1016/s0006-3223(99)00230-9

[CIT0007] Beurel E , Toups M, Nemeroff CB (2020) The bidirectional relationship of depression and inflammation: double trouble. Neuron 107:234–256. 32553197 10.1016/j.neuron.2020.06.002 PMC7381373

[CIT0008] Bobo WV , Vande Voort JL, Croarkin PE, Leung JG, Tye SJ, Frye MA (2016) Ketamine for treatment-resistant unipolar and bipolar major depression: critical review and implications for clinical practice. Depress Anxiety 33:698–710. 27062450 10.1002/da.22505

[CIT0009] Borsini A , Alboni S, Horowitz MA, Tojo LM, Cannazza G, Su KP, Pariante CM, Zunszain PA (2017) Rescue of IL-1β-induced reduction of human neurogenesis by omega-3 fatty acids and antidepressants. Brain Behav Immun 65:230–238. 28529072 10.1016/j.bbi.2017.05.006 PMC5540223

[CIT0010] Borsini A , Cattaneo A, Malpighi C, Thuret S, Harrison NA, Zunszain PA, Pariante CM; MRC ImmunoPsychiatry Consortium (2018) Interferon-alpha reduces human hippocampal neurogenesis and increases apoptosis via activation of distinct STAT1-dependent mechanisms. Int J Neuropsychopharmacol 21:187–200. 29040650 10.1093/ijnp/pyx083 PMC5793815

[CIT0011] Borsini A , Pariante CM, Zunszain PA, Hepgul N, Russell A, Zajkowska Z, Mondelli V, Thuret S (2019) The role of circulatory systemic environment in predicting interferon-alpha-induced depression: the neurogenic process as a potential mechanism. Brain Behav Immun 81:220–227. 31207337 10.1016/j.bbi.2019.06.018 PMC6934231

[CIT0012] Borsini A , Di Benedetto MG, Giacobbe J, Pariante CM (2020a) Pro- and anti-inflammatory properties of interleukin (IL6) in vitro: relevance for major depression and for human hippocampal neurogenesis. Int J Neuropsychopharmacol 23:738–750. 32726406 10.1093/ijnp/pyaa055 PMC7745251

[CIT0013] Borsini A , Stangl D, Jeffries AR, Pariante CM, Thuret S (2020b) The role of omega-3 fatty acids in preventing glucocorticoid-induced reduction in human hippocampal neurogenesis and increase in apoptosis. Transl Psychiatry 10:219. 32636362 10.1038/s41398-020-00908-0 PMC7341841

[CIT0014] Borsini A , Nicolaou A, Camacho-Muñoz D, Kendall AC, Di Benedetto MG, Giacobbe J, Su KP, Pariante CM (2021) Omega-3 polyunsaturated fatty acids protect against inflammation through production of LOX and CYP450 lipid mediators: relevance for major depression and for human hippocampal neurogenesis. Mol Psychiatry 26:6773–6788. 34131267 10.1038/s41380-021-01160-8 PMC8760043

[CIT0015] Borsini A , Merrick B, Edgeworth J, Mandal G, Srivastava DP, Vernon AC, Nebbia G, Thuret S, Pariante CM (2022) Neurogenesis is disrupted in human hippocampal progenitor cells upon exposure to serum samples from hospitalized COVID-19 patients with neurological symptoms. Mol Psychiatry 27:5049–5061. 36195636 10.1038/s41380-022-01741-1 PMC9763123

[CIT0016] Castro-Portuguez R , Sutphin GL (2020) Kynurenine pathway, NAD(+) synthesis, and mitochondrial function: Targeting tryptophan metabolism to promote longevity and healthspan. Exp Gerontol 132:110841. 31954874 10.1016/j.exger.2020.110841 PMC7053056

[CIT0017] Cattane N , Mora C, Lopizzo N, Borsini A, Maj C, Pedrini L, Rossi R, Riva MA, Pariante CM, Cattaneo A (2019) Identification of a miRNAs signature associated with exposure to stress early in life and enhanced vulnerability for schizophrenia: New insights for the key role of miR-125b-1-3p in neurodevelopmental processes. Schizophr Res 205:63–75. 30057098 10.1016/j.schres.2018.07.030

[CIT0018] Cattaneo A , Gennarelli M, Uher R, Breen G, Farmer A, Aitchison KJ, Craig IW, Anacker C, Zunsztain PA, McGuffin P, Pariante CM (2013) Candidate genes expression profile associated with antidepressants response in the GENDEP study: differentiating between baseline “predictors” and longitudinal “targets.” Neuropsychopharmacology 38:377–385. 22990943 10.1038/npp.2012.191 PMC3547188

[CIT0019] Cattaneo A , Cattane N, Malpighi C, Czamara D, Suarez A, Mariani N, Kajantie E, Luoni A, Eriksson JG, Lahti J, Mondelli V, Dazzan P, Räikkönen K, Binder EB, Riva MA, Pariante CM (2018) FoxO1, A2M, and TGF-β1: three novel genes predicting depression in gene X environment interactions are identified using cross-species and cross-tissues transcriptomic and miRNomic analyses. Mol Psychiatry 23:2192–2208. 29302075 10.1038/s41380-017-0002-4 PMC6283860

[CIT0020] Cattaneo A , et al; Neuroimmunology of Mood Disorders and Alzheimer’s Disease (NIMA) Consortium (2020a) Whole-blood expression of inflammasome- and glucocorticoid-related mRNAs correctly separates treatment-resistant depressed patients from drug-free and responsive patients in the BIODEP study. Transl Psychiatry 10:232. 32699209 10.1038/s41398-020-00874-7 PMC7376244

[CIT0021] Cattaneo A , et al; Neuroimmunology of Mood Disorders and Alzheimer’s Disease (NIMA) Consortium (2020b) Correction: whole-blood expression of inflammasome- and glucocorticoid-related mRNAs correctly separates treatment-resistant depressed patients from drug-free and responsive patients in the BIODEP study. Transl Psychiatry 10:352. 33077715 10.1038/s41398-020-01044-5 PMC7572382

[CIT0022] Chang L , Zhang K, Pu Y, Qu Y, Wang SM, Xiong Z, Ren Q, Dong C, Fujita Y, Hashimoto K (2019) Comparison of antidepressant and side effects in mice after intranasal administration of (R,S)-ketamine, (R)-ketamine, and (S)-ketamine. Pharmacol Biochem Behav 181:53–59. 31034852 10.1016/j.pbb.2019.04.008

[CIT0023] Chen MH , Li CT, Lin WC, Hong CJ, Tu PC, Bai YM, Cheng CM, Su TP (2018) Rapid inflammation modulation and antidepressant efficacy of a low-dose ketamine infusion in treatment-resistant depression: a randomized, double-blind control study. Psychiatry Res 269:207–211. 30153598 10.1016/j.psychres.2018.08.078

[CIT0024] Clarke M , Razmjou S, Prowse N, Dwyer Z, Litteljohn D, Pentz R, Anisman H, Hayley S (2017) Ketamine modulates hippocampal neurogenesis and pro-inflammatory cytokines but not stressor induced neurochemical changes. Neuropharmacology 112:210–220. 27106168 10.1016/j.neuropharm.2016.04.021

[CIT0025] Das R , Emon MPZ, Shahriar M, Nahar Z, Islam SMA, Bhuiyan MA, Islam SN, Islam MR (2021) Higher levels of serum IL-1β and TNF-α are associated with an increased probability of major depressive disorder. Psychiatry Res 295:113568. 33199026 10.1016/j.psychres.2020.113568

[CIT0026] DeGiosio RA , Grubisha MJ, MacDonald ML, McKinney BC, Camacho CJ, Sweet RA (2022) More than a marker: potential pathogenic functions of MAP2. Front Mol Neurosci 15:974890. 36187353 10.3389/fnmol.2022.974890 PMC9525131

[CIT0027] Diazgranados N , Ibrahim L, Brutsche NE, Newberg A, Kronstein P, Khalife S, Kammerer WA, Quezado Z, Luckenbaugh DA, Salvadore G, Machado-Vieira R, Manji HK, Zarate CA Jr (2010) A randomized add-on trial of an N-methyl-D-aspartate antagonist in treatment-resistant bipolar depression. Arch Gen Psychiatry 67:793–802. 20679587 10.1001/archgenpsychiatry.2010.90 PMC3000408

[CIT0028] Dong C , Rovnaghi CR, Anand KJ (2012) Ketamine alters the neurogenesis of rat cortical neural stem progenitor cells. Crit Care Med 40:2407–2416. 22635046 10.1097/CCM.0b013e318253563c PMC3507468

[CIT0029] Fischer CW , Eskelund A, Budac DP, Tillmann S, Liebenberg N, Elfving B, Wegener G (2015) Interferon-alpha treatment induces depression-like behaviour accompanied by elevated hippocampal quinolinic acid levels in rats. Behav Brain Res 293:166–172. 26205824 10.1016/j.bbr.2015.07.015

[CIT0030] Fricker RA , Green EL, Jenkins SI, Griffin SM (2018) The influence of nicotinamide on health and disease in the central nervous system. Int J Tryptophan Res 11:1178646918776658. 29844677 10.1177/1178646918776658 PMC5966847

[CIT0031] Fukumoto K , Toki H, Iijima M, Hashihayata T, Yamaguchi JI, Hashimoto K, Chaki S (2017) Antidepressant potential of (R)-ketamine in rodent models: comparison with (S)-ketamine. J Pharmacol Exp Ther 361:9–16. 28115553 10.1124/jpet.116.239228

[CIT0032] Gómez-Climent MA , Castillo-Gómez E, Varea E, Guirado R, Blasco-Ibáñez JM, Crespo C, Martínez-Guijarro FJ, Nácher J (2008) A population of prenatally generated cells in the rat paleocortex maintains an immature neuronal phenotype into adulthood. Cereb Cortex 18:2229–2240. 18245040 10.1093/cercor/bhm255

[CIT0033] Hack LM , Provençal N, Wiechmann T, Koedel M, Rex-Haffner M, Anacker C, Binder E (2016) Exposure to glucocorticoids during hippocampal neurogenesis: effects on DNA hydroxymethylation. Biol Psychiatry 79:94S–94S.

[CIT0034] Hepgul N , Cattaneo A, Agarwal K, Baraldi S, Borsini A, Bufalino C, Forton DM, Mondelli V, Nikkheslat N, Lopizzo N, Riva MA, Russell A, Hotopf M, Pariante CM (2016) Transcriptomics in interferon-α-treated patients identifies inflammation-, neuroplasticity- and oxidative stress-related signatures as predictors and correlates of depression. Neuropsychopharmacology 41:2502–2511. 27067128 10.1038/npp.2016.50 PMC4983179

[CIT0035] Horowitz MA , Cattaneo A, Cattane N, Lopizzo N, Tojo L, Bakunina N, Musaelyan K, Borsini A, Zunszain PA, Pariante CM (2020) Glucocorticoids prime the inflammatory response of human hippocampal cells through up-regulation of inflammatory pathways. Brain Behav Immun 87:777–794. 32194233 10.1016/j.bbi.2020.03.012

[CIT0036] Jelen LA , Young AH, Stone JM (2021) Ketamine: a tale of two enantiomers. J Psychopharmacol 35:109–123. 33155503 10.1177/0269881120959644 PMC7859674

[CIT0037] Kadriu B , Farmer CA, Yuan P, Park LT, Deng ZD, Moaddel R, Henter ID, Shovestul B, Ballard ED, Kraus C, Gold PW, Machado-Vieira R, Zarate CA Jr (2021) The kynurenine pathway and bipolar disorder: intersection of the monoaminergic and glutamatergic systems and immune response. Mol Psychiatry 26:4085–4095. 31732715 10.1038/s41380-019-0589-8 PMC7225078

[CIT0038] Kim H , Chen L, Lim G, Sung B, Wang S, McCabe MF, Rusanescu G, Yang L, Tian Y, Mao J (2012) Brain indoleamine 2,3-dioxygenase contributes to the comorbidity of pain and depression. J Clin Invest 122:2940–2954. 22751107 10.1172/JCI61884 PMC3408737

[CIT0039] Kiraly DD , Horn SR, Van Dam NT, Costi S, Schwartz J, Kim-Schulze S, Patel M, Hodes GE, Russo SJ, Merad M, Iosifescu DV, Charney DS, Murrough JW (2017) Altered peripheral immune profiles in treatment-resistant depression: response to ketamine and prediction of treatment outcome. Transl Psychiatry 7:e1065. 28323284 10.1038/tp.2017.31 PMC5416674

[CIT0040] Kopra E , Mondelli V, Pariante C, Nikkheslat N (2021) Ketamine’s effect on inflammation and kynurenine pathway in depression: a systematic review. J Psychopharmacol 35:934–945. 34180293 10.1177/02698811211026426 PMC8358579

[CIT0041] Kuzior H , Fiebich BL, Yousif NM, Saliba SW, Ziegler C, Nickel K, Maier SJ, Süß P, Runge K, Matysik M, Dersch R, Berger B, Robinson T, Venhoff N, Kessler F, Blank T, Domschke K, Tebartz van Elst L, Endres D (2020) Increased IL-8 concentrations in the cerebrospinal fluid of patients with unipolar depression. Compr Psychiatry 102:152196. 32927367 10.1016/j.comppsych.2020.152196

[CIT0042] Kwidzinski E , Bechmann I (2007) IDO expression in the brain: a double-edged sword. J Mol Med (Berl) 85:1351–1359. 17594069 10.1007/s00109-007-0229-7

[CIT0043] Loix S , De Kock M, Henin P (2011) The anti-inflammatory effects of ketamine: state of the art. Acta Anaesthesiol Belg 62:47–58. 21612145

[CIT0044] Lugo-Huitrón R , Ugalde Muñiz P, Pineda B, Pedraza-Chaverrí J, Ríos C, Pérez-de la Cruz V (2013) Quinolinic acid: an endogenous neurotoxin with multiple targets. Oxid Med Cell Longev 2013:104024. 24089628 10.1155/2013/104024 PMC3780648

[CIT0045] Matveychuk D , Thomas RK, Swainson J, Khullar A, MacKay MA, Baker GB, Dursun SM (2020) Ketamine as an antidepressant: overview of its mechanisms of action and potential predictive biomarkers. Ther Adv Psychopharmacol 10:2045125320916657. 32440333 10.1177/2045125320916657 PMC7225830

[CIT0046] Murrough JW , Iosifescu DV, Chang LC, Al Jurdi RK, Green CE, Perez AM, Iqbal S, Pillemer S, Foulkes A, Shah A, Charney DS, Mathew SJ (2013) Antidepressant efficacy of ketamine in treatment-resistant major depression: a two-site randomized controlled trial. Am J Psychiatry 170:1134–1142. 23982301 10.1176/appi.ajp.2013.13030392 PMC3992936

[CIT0047] Nettis MA , Lombardo G, Hastings C, Zajkowska Z, Mariani N, Nikkheslat N, Worrell C, Enache D, McLaughlin A, Kose M, Sforzini L, Bogdanova A, Cleare A, Young AH, Pariante CM, Mondelli V (2021) Augmentation therapy with minocycline in treatment-resistant depression patients with low-grade peripheral inflammation: results from a double-blind randomised clinical trial. Neuropsychopharmacology 46:939–948. 33504955 10.1038/s41386-020-00948-6 PMC8096832

[CIT0048] Newport DJ , Carpenter LL, McDonald WM, Potash JB, Tohen M, Nemeroff CB; APA Council of Research Task Force on Novel Biomarkers and Treatments (2015) Ketamine and other NMDA antagonists: early clinical trials and possible mechanisms in depression. Am J Psychiatry 172:950–966. 26423481 10.1176/appi.ajp.2015.15040465

[CIT0049] Nikkheslat N (2021) Targeting inflammation in depression: ketamine as an anti-inflammatory antidepressant in psychiatric emergency. Brain Behav Immun Health 18:100383. 34849492 10.1016/j.bbih.2021.100383 PMC8609146

[CIT0050] Nikolin S , Rodgers A, Schwaab A, Bahji A, Zarate C Jr, Vazquez G, Loo C, Loo C (2023) Ketamine for the treatment of major depression: a systematic review and meta-analysis. EClinicalMedicine 62:102127. 37593223 10.1016/j.eclinm.2023.102127 PMC10430179

[CIT0051] O’Connor JC , André C, Wang Y, Lawson MA, Szegedi SS, Lestage J, Castanon N, Kelley KW, Dantzer R (2009) Interferon-gamma and tumor necrosis factor-alpha mediate the upregulation of indoleamine 2,3-dioxygenase and the induction of depressive-like behavior in mice in response to bacillus Calmette-Guerin. J Neurosci 29:4200–4209. 19339614 10.1523/JNEUROSCI.5032-08.2009 PMC2835569

[CIT0052] Osimo EF , Pillinger T, Rodriguez IM, Khandaker GM, Pariante CM, Howes OD (2020) Inflammatory markers in depression: a meta-analysis of mean differences and variability in 5,166 patients and 5,083 controls. Brain Behav Immun 87:901–909. 32113908 10.1016/j.bbi.2020.02.010 PMC7327519

[CIT0053] Passie T , Adams HA, Logemann F, Brandt SD, Wiese B, Karst M (2021) Comparative effects of (S)-ketamine and racemic (R/S)-ketamine on psychopathology, state of consciousness and neurocognitive performance in healthy volunteers. Eur Neuropsychopharmacol 44:92–104. 33487513 10.1016/j.euroneuro.2021.01.005

[CIT0054] Pfaffl MW (2001) A new mathematical model for relative quantification in real-time RT-PCR. Nucleic Acids Res 29:e45. 11328886 10.1093/nar/29.9.e45 PMC55695

[CIT0055] Pitharouli MC , Hagenaars SP, Glanville KP, Coleman JRI, Hotopf M, Lewis CM, Pariante CM (2021) Elevated C-reactive protein in patients with depression, independent of genetic, health, and psychosocial factors: results from the UK biobank. Am J Psychiatry 178:522–529. 33985349 10.1176/appi.ajp.2020.20060947

[CIT0056] Santarelli L , Saxe M, Gross C, Surget A, Battaglia F, Dulawa S, Weisstaub N, Lee J, Duman R, Arancio O, Belzung C, Hen R (2003) Requirement of hippocampal neurogenesis for the behavioral effects of antidepressants. Science 301:805–809. 12907793 10.1126/science.1083328

[CIT0057] Savitz J (2020) The kynurenine pathway: a finger in every pie. Mol Psychiatry 25:131–147. 30980044 10.1038/s41380-019-0414-4 PMC6790159

[CIT0058] Savitz J , Drevets WC, Smith CM, Victor TA, Wurfel BE, Bellgowan PS, Bodurka J, Teague TK, Dantzer R (2015) Putative neuroprotective and neurotoxic kynurenine pathway metabolites are associated with hippocampal and amygdalar volumes in subjects with major depressive disorder. Neuropsychopharmacology 40:463–471. 25074636 10.1038/npp.2014.194 PMC4443961

[CIT0059] Strawbridge R , Arnone D, Danese A, Papadopoulos A, Herane Vives A, Cleare AJ (2015) Inflammation and clinical response to treatment in depression: a meta-analysis. Eur Neuropsychopharmacol 25:1532–1543. 26169573 10.1016/j.euroneuro.2015.06.007

[CIT0060] Strong CE , Kabbaj M (2018) On the safety of repeated ketamine infusions for the treatment of depression: Effects of sex and developmental periods. Neurobiol Stress 9:166–175. 30450382 10.1016/j.ynstr.2018.09.001 PMC6236511

[CIT0061] Su KP , Lai HC, Peng CY, Su WP, Chang JP, Pariante CM (2019) Interferon-alpha-induced depression: Comparisons between early- and late-onset subgroups and with patients with major depressive disorder. Brain Behav Immun 80:512–518. 31059806 10.1016/j.bbi.2019.04.032

[CIT0062] Suhee FI , Shahriar M, Islam SMA, Bhuiyan MA, Islam MR (2023) Elevated serum IL-2 levels are associated with major depressive disorder: a case-control study. Clin Pathol 16:2632010–x231180797. 10.1177/2632010X231180797 PMC10285590 37360518

[CIT0063] Sukhram SD , Yilmaz G, Gu J (2022) Antidepressant effect of ketamine on inflammation-mediated cytokine dysregulation in adults with treatment-resistant depression: rapid systematic review. Oxid Med Cell Longev 2022:1061274. 36160713 10.1155/2022/1061274 PMC9507757

[CIT0064] Tuglu C , Kara SH, Caliyurt O, Vardar E, Abay E (2003) Increased serum tumor necrosis factor-alpha levels and treatment response in major depressive disorder. Psychopharmacology (Berl) 170:429–433. 12955291 10.1007/s00213-003-1566-z

[CIT0065] Vai B , Mazza MG, Cazzetta S, Calesella F, Aggio V, Lorenzi C, Zanardi R, Poletti S, Colombo C, Benedetti F (2021) Higher Interleukin 13 differentiates patients with a positive history of suicide attempts in major depressive disorder. J Affect Dis Reports 6:100254.

[CIT0066] Wu A , Zhang J (2023) Neuroinflammation, memory, and depression: new approaches to hippocampal neurogenesis. J Neuroinflammation 20:283. 38012702 10.1186/s12974-023-02964-x PMC10683283

[CIT0067] Wu GH , Guo QH, Xu XD, Lin JC, You GT, Lin CH, Zhang LC (2023) Ketamine exerts dual effects on the apoptosis of primary cultured hippocampal neurons from fetal rats in vitro. Metab Brain Dis 38:2417–2426. 37273081 10.1007/s11011-023-01236-0

[CIT0068] Yang C , Hong T, Shen J, Ding J, Dai XW, Zhou ZQ, Yang JJ (2013a) Ketamine exerts antidepressant effects and reduces IL-1β and IL-6 levels in rat prefrontal cortex and hippocampus. Exp Ther Med 5:1093–1096. 23596475 10.3892/etm.2013.930 PMC3627439

[CIT0069] Yang C , Shen J, Hong T, Hu TT, Li ZJ, Zhang HT, Zhang YJ, Zhou ZQ, Yang JJ (2013b) Effects of ketamine on lipopolysaccharide-induced depressive-like behavior and the expression of inflammatory cytokines in the rat prefrontal cortex. Mol Med Rep 8:887–890. 23900245 10.3892/mmr.2013.1600

[CIT0070] Yang C , Shirayama Y, Zhang JC, Ren Q, Yao W, Ma M, Dong C, Hashimoto K (2015a) R-ketamine: a rapid-onset and sustained antidepressant without psychotomimetic side effects. Transl Psychiatry 5:e632. 26327690 10.1038/tp.2015.136 PMC5068814

[CIT0071] Yang C , Wardenaar KJ, Bosker FJ, Li J, Schoevers RA (2019) Inflammatory markers and treatment outcome in treatment resistant depression: a systematic review. J Affect Disord 257:640–649. 31357161 10.1016/j.jad.2019.07.045

[CIT0072] Yang JJ , Wang N, Yang C, Shi JY, Yu HY, Hashimoto K (2015b) Serum interleukin-6 is a predictive biomarker for ketamine’s antidepressant effect in treatment-resistant patients with major depression. Biol Psychiatry 77:e19–e20. 25104172 10.1016/j.biopsych.2014.06.021

[CIT0073] Zarate CA Jr , Brutsche N, Laje G, Luckenbaugh DA, Venkata SL, Ramamoorthy A, Moaddel R, Wainer IW (2012) Relationship of ketamine’s plasma metabolites with response, diagnosis, and side effects in major depression. Biol Psychiatry 72:331–338. 22516044 10.1016/j.biopsych.2012.03.004 PMC3442255

[CIT0074] Zhao J , Zhang R, Wang W, Jiang S, Liang H, Guo C, Qi J, Zeng H, Song H (2023) Low-dose ketamine inhibits neuronal apoptosis and neuroinflammation in PC12 cells via α7nAChR mediated TLR4/MAPK/NF-κB signaling pathway. Int Immunopharmacol 117:109880. 36842233 10.1016/j.intimp.2023.109880

[CIT0075] Zhao X , Venkata SL, Moaddel R, Luckenbaugh DA, Brutsche NE, Ibrahim L, Zarate CA Jr, Mager DE, Wainer IW (2012) Simultaneous population pharmacokinetic modelling of ketamine and three major metabolites in patients with treatment-resistant bipolar depression. Br J Clin Pharmacol 74:304–314. 22295895 10.1111/j.1365-2125.2012.04198.x PMC3630750

[CIT0076] Zhou Y , Zheng W, Liu W, Wang C, Zhan Y, Li H, Chen L, Li M, Ning Y (2018) Antidepressant effect of repeated ketamine administration on kynurenine pathway metabolites in patients with unipolar and bipolar depression. Brain Behav Immun 74:205–212. 30213652 10.1016/j.bbi.2018.09.007

[CIT0077] Zhou YL , Wu FC, Wang CY, Zheng W, Lan XF, Deng XR, Ning YP (2020) Relationship between hippocampal volume and inflammatory markers following six infusions of ketamine in major depressive disorder. J Affect Disord 276:608–615. 32871692 10.1016/j.jad.2020.06.068

[CIT0078] Zunszain PA , Anacker C, Cattaneo A, Choudhury S, Musaelyan K, Myint AM, Thuret S, Price J, Pariante CM (2012) Interleukin-1β: a new regulator of the kynurenine pathway affecting human hippocampal neurogenesis. Neuropsychopharmacology 37:939–949. 22071871 10.1038/npp.2011.277 PMC3280640

