# Supplementary figures and images for: Ketamine Prevents Inflammation-Induced Reduction of Human Hippocampal Neurogenesis via Inhibiting the Production of Neurotoxic Metabolites of the Kynurenine Pathway
Source: Int J Neuropsychopharmacol. 2024 Sep 19;27(10):pyae041. doi: 10.1093/ijnp/pyae041 (PMC11450635; doi:10.1093/ijnp/pyae041)

a)

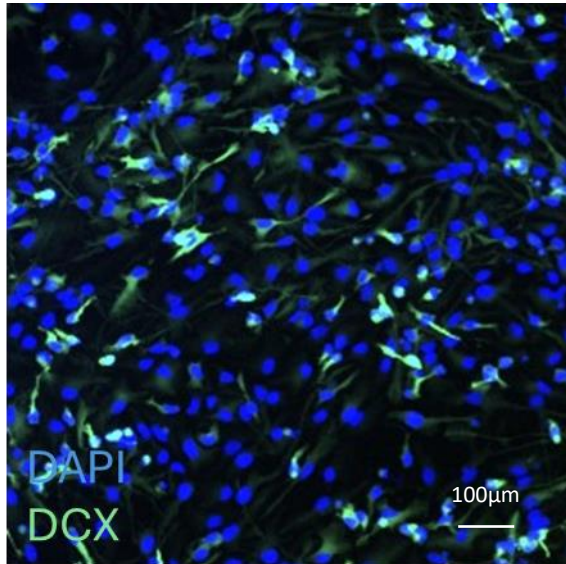

b)

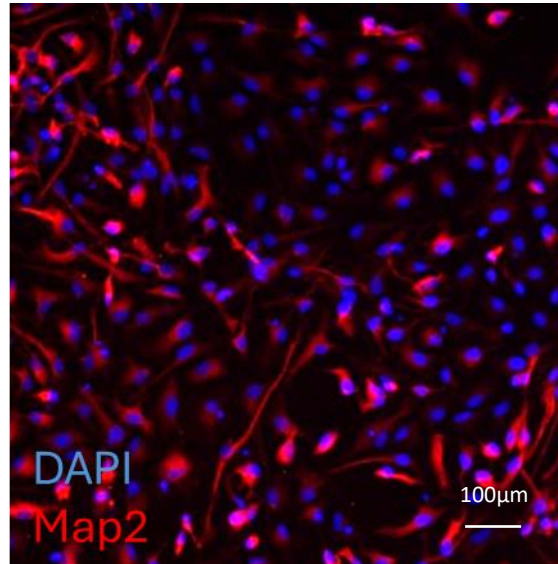

c)

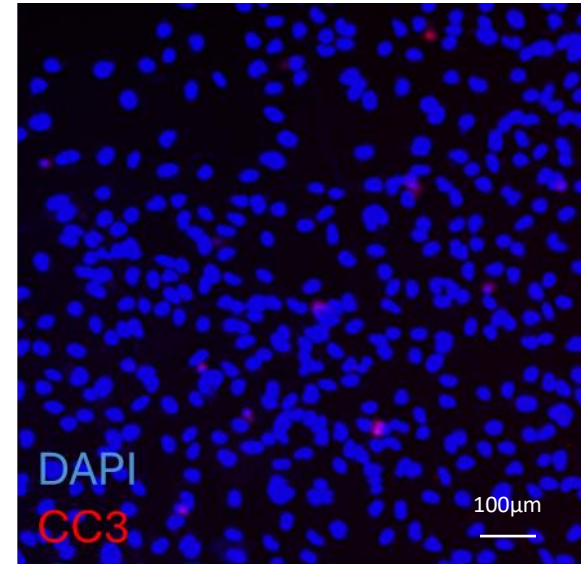

Supplement: pyae041_suppl_Supplementary_Figure_S1 [file pyae041_suppl_supplementary_figure_s1.pdf]

a)

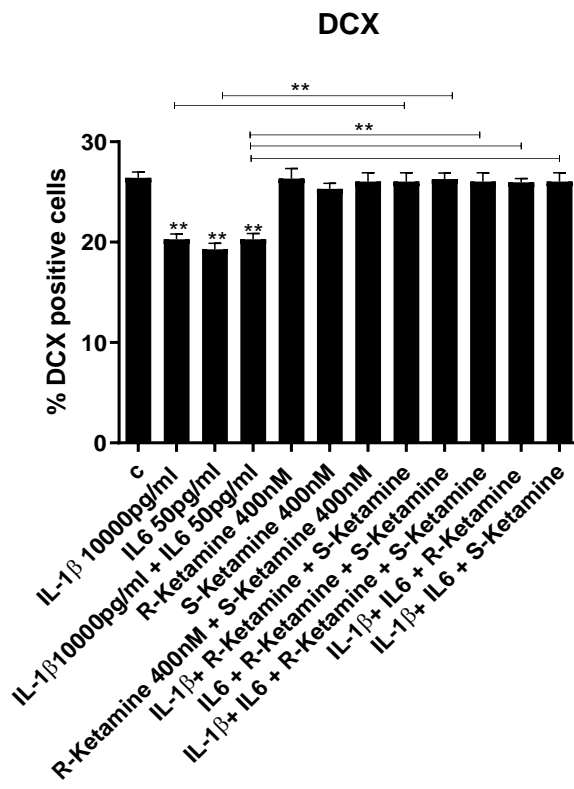

b)

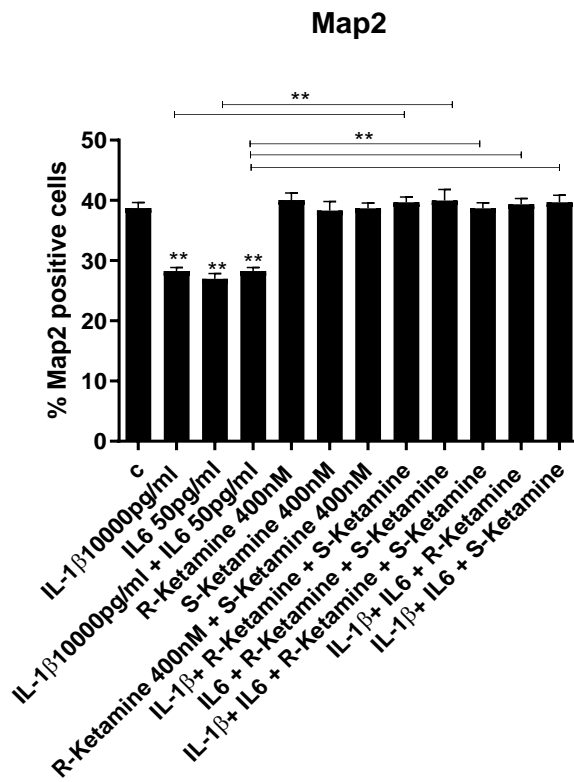

c)

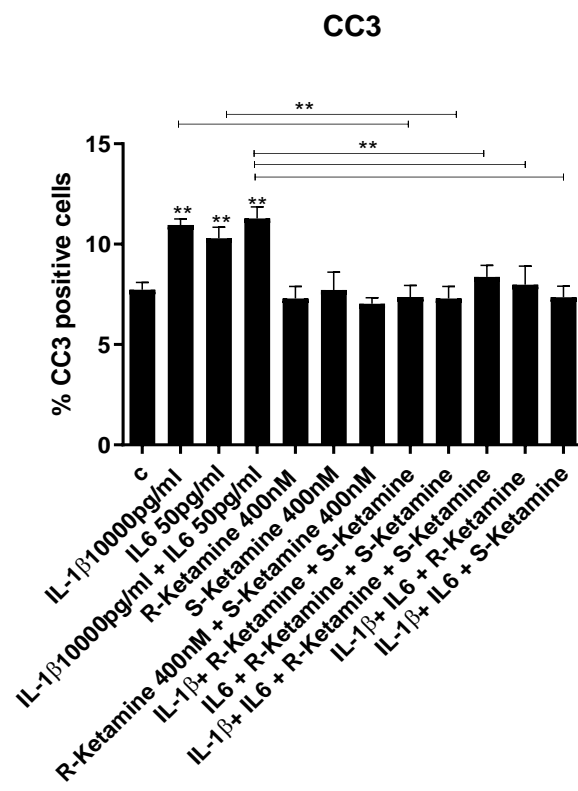

Supplement: pyae041_suppl_Supplementary_Figure_S2 [file pyae041_suppl_supplementary_figure_s2.pdf]
